# Shaping of topography by topographically-controlled vegetation in tropical montane rainforest

**DOI:** 10.1371/journal.pone.0281835

**Published:** 2023-03-09

**Authors:** Gilles Brocard, Jane K. Willebring, Fred N. Scatena

**Affiliations:** Department of Earth and Environmental Sciences, University of Pennsylvania, Philadelphia, Pennsylvania, United States of America; Georgia Southern University, UNITED STATES

## Abstract

Topography is commonly viewed as a passive backdrop on which vegetation grows. Yet, in certain circumstances, a bidirectional feedback may develop between the control of topography and the spatial distribution of vegetation and landform development, because vegetation modulates the erosion of the land surface. Therefore, if reinforcing feedbacks are established between erosion and land cover distribution over timescales relevant to landform development, then the interactions between vegetation and topography may create distinctive landforms, shaped by vegetation. We expose here a strong correlation between the spatial distribution of vegetation, erosion rates, and topography at a characteristic length scale of 10^2^-10^3^m (mesoscale topography) in the Luquillo Experimental forest (LEF) of Puerto Rico. We use high-resolution LiDAR topography to characterize landforms, satellite images to classify the vegetation into forest types, and in-situ produced cosmogenic ^10^Be in the quartz extracted from soils and stream sediments to document spatial variations in soil erosion. The data document a strong correlation between forest type and topographic position (hilltop vs. valleys), and a correlation between topographic position and ^10^Be-derived erosion rates over 10^3^−10^4^ years. Erosion is faster in valleys, which are mostly covered by monocot Palm Forest, and slower on surrounding hills mostly covered by the dicot Palo Colorado Forest. Transition from one forest type to the next occurs across a break-in-slope that separates shallowly convex hilltops from deeply concave valleys (coves). The break-in-slope is the consequence of a longer-lasting erosional imbalance whereby coves erode faster than hills over landscape-shaping timescales. Such a deepening of the coves is usually spurred by external drivers, but such drivers are here absent. This implies that cove erosion is driven by a process originating within the coves themselves. We propose that vegetation is the primary driver of this imbalance, soil erosion being faster under Palm forest than under Palo Colorado forest. Concentration of the Palm forest in the deepening coves is reinforced by the better adaptation of Palm trees to the erosive processes that take place in the coves, once these develop steep slopes. At the current rate of landscape development, we find that the imbalance started within the past 0.1–1.5 My. The initiation of the process could correspond to time of settlement of these mountain slopes by the Palm and Palo Colorado forests.

## Introduction

Topography affects the distribution of vegetation over a wide range of spatial scales. At the scale of mountain ranges (macroscale, 10^4^−10^5^ m), topography affects vegetation through its control on climate, and water availability along slopes [1]. At the scale of hills and valleys (mesoscale, 10^2^−10^4^ m), topography affects the distribution of vegetation through its role in routing water, nutrients, and sediments [2, 3]. Ground cover by vegetation affects soil erosion rates. Some models, therefore, predict that vegetation should, in turn, influence landscape development [4, 5], although it has been difficult, so far, to identify landforms that unequivocally bear the imprint of life [6]. The influence of topographically-controlled vegetation on erosion rates has been documented at the mesoscale (10^2^-10^3^m) [7], albeit over relatively short timescales (10^2^-10^3^y), too short to address their effect on landscape development, or potential feedbacks. Here we document the distribution of landforms, vegetation, and erosion rates across the tropical montane forest of Luquillo, in Puerto Rico, over timescales relevant to landscape development.

The study relies on three datasets: a high-resolution LiDAR digital elevation model (DEM), high-resolution satellite images, and in situ-produced cosmogenic ^10^Be measurements in quartz collected in forest soils and river sands. The DEM and the satellite images are used to explore correlations between vegetation and topography. The ^10^Be data is combined to the DEM data to analyze the distribution of erosion rates across the topography over 10^3^−10^4^ years. The geomorphological analysis of the DEM is used to identify landforms produced by longer (10^4^−10^6^ years) imbalance in erosion across the landscape. The current imbalance in ^10^Be-derived erosion rates is used to calculate the time required to produce the current landscape over the timescale revealed by the DEM analysis. We then review the potential drivers of observed spatial variations in erosion rates. We propose that the variations are strongly influenced by the distribution of vegetation across the landscape.

**Fig 1:**
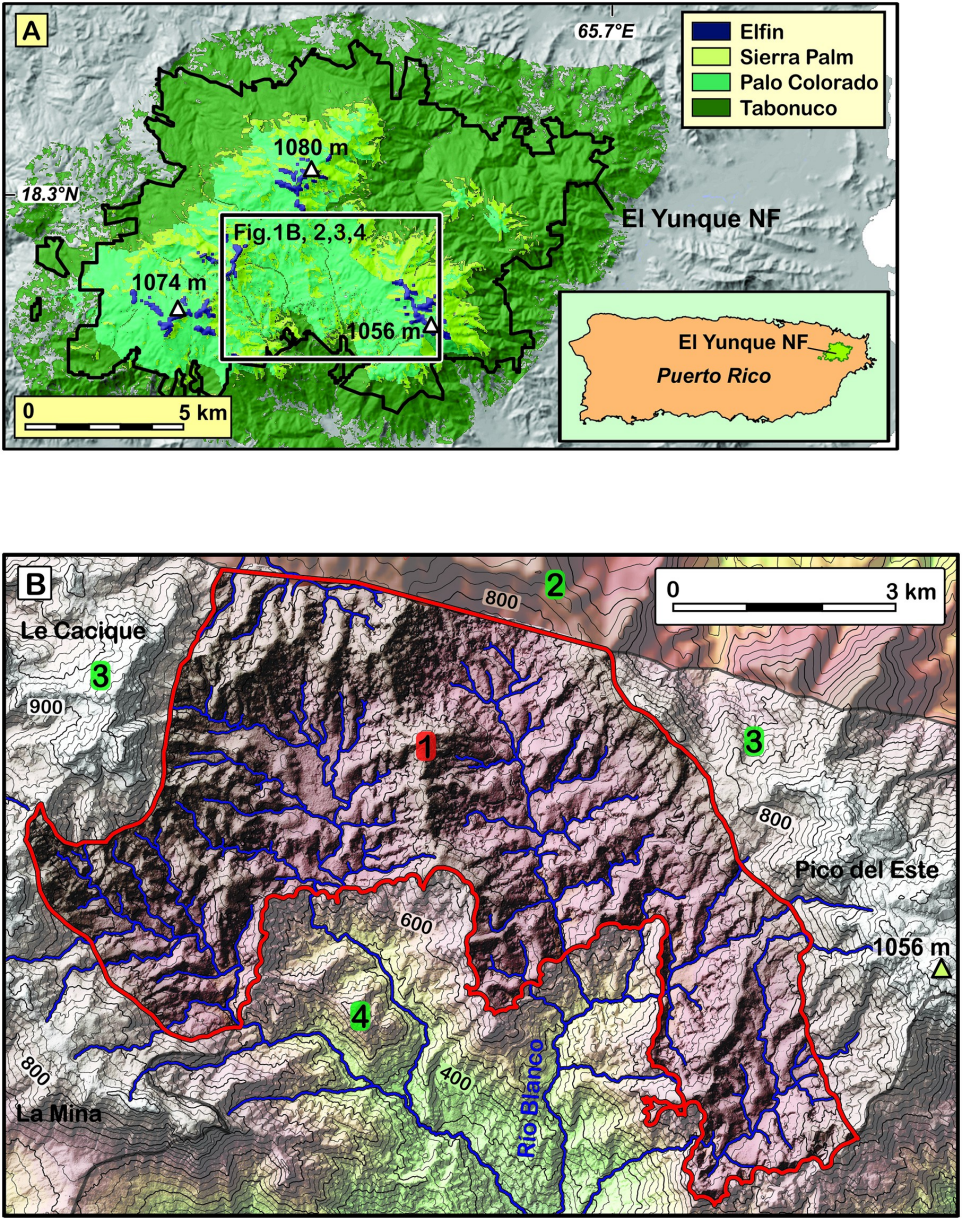
Geographic setting of the study area. A: Map showing the altitudinal zoning of vegetation that characterizes the El Yunque National Forest, over a shaded relief of the Luquillo Mountains. Black outline: border of El Yunque National Forest. Inset: Location of the forest on Puerto Rico island. Vegetation data from (Gould et al., 2008) [14]. Tabonuco Forest: tall lower montane rainforest, Sierra Palm Forest and Palo Colorado Forest: monocot and dicot-dominated montane rainforests, Elfin: dwarfed forest on mountain tops. B: topographic map showing the limits (red line) of the studied area (1). The extent of the study area was determined by exclusion of areas lacking either LiDAR coverage (2) or the quartz necessary for ^10^Be-based quantification of erosion rates (3), and by exclusion of areas affected by accelerated erosion (4).

The study area covers 16.3 km^2^ in the headwaters of Río Blanco, a river that drains the southern flank of the Luquillo Mountains in Puerto Rico (Fig 1A). The Río Blanco headwaters are part of the El Yunque National Forest, and of the Luquillo Experimental Forest (LEF). Vegetation in the Luquillo mountains is strongly zoned according to elevation (Fig 1A). Forest types include lower montane Tabonuco forest below 600 m, cloud (“elfin”) forest above 950 m (Fig 1A). In between, the two dominant forest types are the Sierra Palm (*Prestoea Montana*) forest and the Palo Colorado (*Cyrilla Racemifolia*) forest, which respectively cover 43% and 57% of the study area, located between 600 and 750 m.

The study area (Fig 1B) features 200–800 m-wide hills composed of shallow-sloping, low-curvature tops, flanked by steep slopes (up to >30°) above first-order stream valleys. These valleys are typical of mountain coves, with short lengths and strongly concave-up cross-sections. The local relief (defined as the altitudinal difference between valley floors and surrounding hilltops) is 145 ± 30 m on average, and nowhere exceeds 225 m. Precipitation is very frequent in the Luquillo Mountains, such that the coves are drained by permanent creeks. These creeks drain into higher-order, shallow-gradient, meandering streams which are surrounded by 50- to 150 m-wide sandy to gravelly floodplains. The a bedrock below the study area is composed of quartz diorite, which provides spatially-homogeneous, and abundant source of quartz (23% by mass [8]), which is so ideal for the quantification of spatial variations in erosion rates that it was chosen in the past to test the validity of the detrital ^10^Be method for quantifying erosion rates [9]. The stock of quartz diorite is surrounded by a resistant aureole of quartz-poor hornfelsed volcanoclastic basalts and basaltic andesites which support the highest peaks of the Luquillo Mountains. The study area lies upstream of a deeply dissected landscape, formed by the propagation of an erosion wave along the lower reaches of Río Blanco [10], which was excluded from the study, as the forest types that cover these slopes do not show the clear influence of topography find farther upstream. Despite relatively steep slopes (23 ± 10°, 1σ) and high precipitation (up to 5,000 mm/y [11]), the study area erodes slowly, at 40–60 m/My [9, 12], presumably owing to the protective role of the forest cover [10].

## Materials and methods

### LiDAR data processing

Airborne LiDAR data were collected by the National Centre for Airborne Laser Mapping (NCALM) in May 2011 (S1 File). Persistent cloudiness over some parts of the mountains prevented reaching a point cloud density sufficient to reliably identify ground returns [13]. This affect only 3% of the study area, however, and that part was patched with a 10 m-resolution DEM. The 1 m-resolution LiDAR DEM was resampled at 5 m to reduce noise resulting from the inability of algorithms to tease out the scattering resulting from the low density of ground returns from the scattering produced by the presence of large corestones at the bottom of many coves (S1 Grid 1 in S1 File). The 5m-resolution DEM was used to calculate topographic metrics that capture some of the topographic characteristics that affect the distribution of vegetation: elevation, aspect, slope steepness, and depth between two consecutive ridgetops (hereafter referred to as entrenchment depth). This later parameter was obtained by passing a surface envelope through the hilltops, and then subtracting the 5m-resolution DEM from this envelope (S1, S1 Grid 2 in S1 File).

### Image processing: Forest classification

A cloudless, high resolution satellite image was obtained by combining cloudy multispectral images (S2 File). The studied area consists of 16.3 km^2^ of forest located within the boundaries of the reliable LiDAR data [13], above the upper limit of the Tabonuco forest [14]. As the analysis focused on the interactions topography and vegetation over areas underlain by the bedrock of quartz-releasing rocks, other landscape elements were excluded, in particular water-logged, sedimented sandy floodplains [15], and aprons of volcanoclastic gravel stemming from surrounding mountain peaks. Canopy-forming vegetation was grouped into the two dominant forest types, namely the Palo Colorado and Palm forests, by supervised classification with ArcGIS 9, based on the distinctive spectral signatures of these forests on the 2 m-resolution multispectral images. Training areas where chosen on 40 cm-resolution panchromatic images acquired at the same time as the multispectral images. Selection was based on the distinctively different textures of these two forest types, and were ground-proofed. The classified image (S2 Grid 1) was used to calculate pixel-to pixel correlations between topographic metrics and forest types, over 11.5 km^2^ of the classified forest (S2 Table 1 in S2 File).

### Calculation of in situ ^10^Be-derived erosion rates

**Fig 2:**
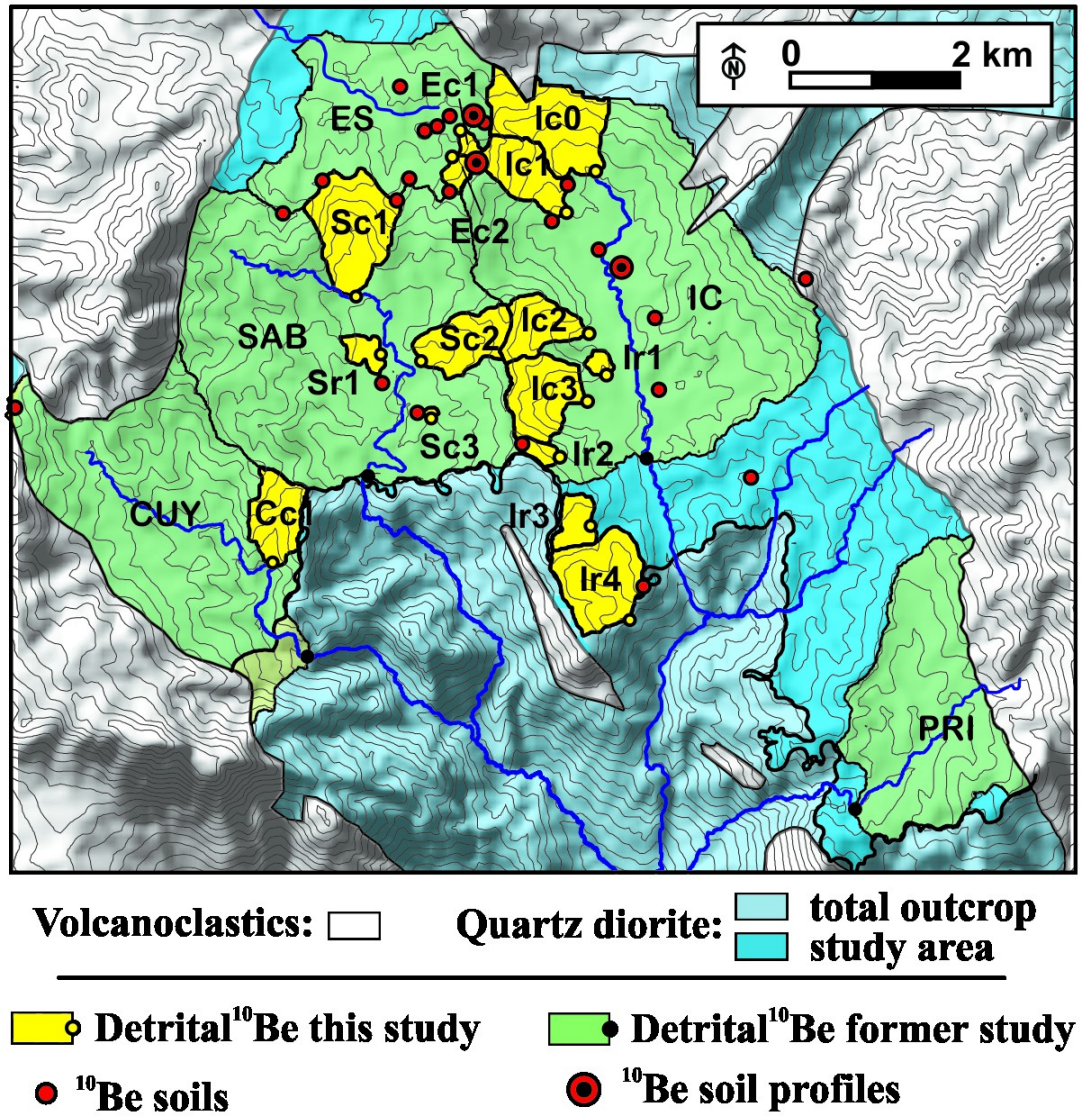
Sampling locations chosen for ^10^Be measurements in soils and river sediments. The map shows the extent of the watershed feeding river borne quartz used for ^10^Be detrital rates measurements, in this study and earlier studies [9, 12]. Grey lines: contour lines with 25 m elevation spacing (10 m-resolution DEM). Large catchment names: CUY: Cubuy, ES: Espiritú Santo, IC: Icacos, PRI: Prieto, SAB: Sabana.

Terrestrial cosmogenic ^10^Be is produced in quartz as quartz minerals are exhumed from the topmost few meters below the ground surface. The concentration of ^10^Be therefore depends on the residence time of quartz within these top meters, residence time which is a function of the rate at which the surface is eroded. ^10^Be concentration in a hilltop soil is simply a function the upward flux of quartz to the surface. From there, soil particle fluxes diverge laterally away from the hilltop toward the hill flanks. We obtained site-specific denudation rates from 27 surface soils samples taken at widely distributed hilltop locations (Fig 2, and S3 Table 3 in S3 File). They provide reliable denudation rates if erosion is close to steady, over the duration of ^10^Be build-up. We tested the validity of this assumption at three sites, by measuring the decline of the ^10^Be concentration with depth below the ground surface (S3 Table 1 and S3 Fig 1 in S3 File).

The measurement of erosion rates in a hillslope is made more complex by the fact that particle fluxes in soils also incorporate flux of soil particles from upslope, resulting in the mixing of vertical and lateral fluxes. Other compounding factors include the fact that, while in the case at hand the broad, shallow-sloping hilltops are eroded mainly by diffusive processes, slopes undergo more unsteady owing to the occurrence of landsides with a return period of about 10 ky [16, 17]. Besides, slopes are frequently chocked with large corestones toward their toe. The corestones constitute large transient topographic obstructions to cosmic rays which make ^10^Be production irregular over time. The inventory of in situ produced ^10^Be in stream sediments therefore provides an alternate approach, which commonly used to retrieve denudation rates averaged over the contributing catchment. The study area was the site of pioneering study where this approach was successfully tested [9]. To contrast the erosion of shallow-sloping, low-curvature hilltops to that of the coves, we selected rivers spanning a broad apportionment of hilltops and coves. We sampled river bedload, extracted the quartz fraction, and measured ^10^Be in the river-borne quartz (S3 Table 4 in S3 File). Usually, erosion rates are calculated using the ^10^Be concentration in only one grain-size (250–500 μm in most cases). However, the more thorough investigations conducted in the study area have shown that ^10^Be concentration there varies with sediment grain size [9, 12]. This property has been attributed to landsliding [9], or to systematic spatial variations in quartz exhumation conditions [12]. Regardless of its causes, this dependency introduces some uncertainty into the conversion of ^10^Be concentrations into basin-wide erosion rates. Therefore, in order to capture the full range of apparent denudation rates introduced by this dependency, we measured the ^10^Be concentration in the finest and coarsest measurable sediment grain size fractions across 14 streams, wherever such fractions stood at least six phi units apart (S3.3.2.3 in S3 File). Only the fine fraction was measured otherwise (S3 Table 4 in S3 File). We then conducted a sensitivity analysis of the effect of sediment grain size on erosion rates, and on other calculations further derived from these erosion rates (S3 Table 2 and S3 Fig 2 in S3 File). All samples were prepared at the University of Pennsylvania Cosmogenic Isotope Laboratory (PennCIL) and measured at the PRIME lab, Purdue University, Indiana.

## Results

### Landform characteristics from LiDAR data

**Fig 3:**
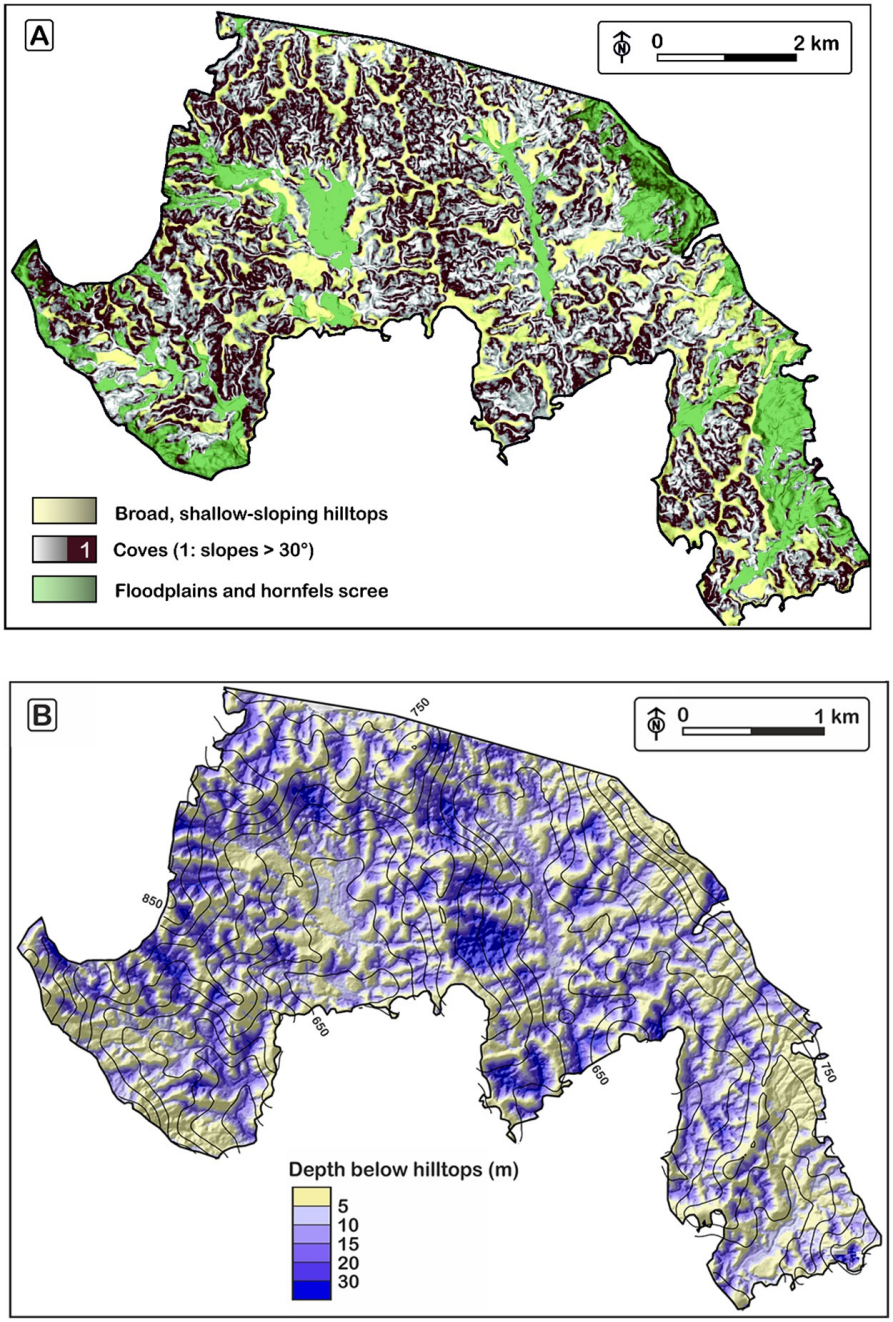
Maps of the mesoscale topography. A: Map of slopes steeper than the landslide threshold (30°), used to delineate cove walls. HF: hornfels (silicified volcanoclastics). B: Depth of incision of the coves below the shallower landscape represented by broad low-curvature hilltops (yellow). Black solid contour lines (spacing 25m): elevation of the surface enveloping the broad hilltops.

Within the study area, ridgelines possess shallow slopes along strike, and shallow crosswise slopes (< 15°, Fig 3A). They also display shallow, convex-up curvature (< 0.05 m^-1^). They are systematically separated from surrounding coves by a break-in-slope, below which slopes reach values ≥ 30° (Fig 3A). This value is the local threshold beyond which landsliding occurs [17]. Below the break-in-slope, the coves are characterized by steep, concave-up, amphitheater-shaped valley heads. At places where valley heads touch one another, only thin knife-edge ridges are present. The break-in-slope is not related to any change in underlying soil or rock resistance, and therefore result from an imbalance in erosion rates, between slowly eroding upper slopes and faster eroding lower slopes. The present-day surface, substracted from a surface envelope interpolated over the broad ridgelines (see S1 File, and contours on Fig 3B) provides a map of the depth of entrenchment of the coves between the surrounding hilltops, below the break-in-slope (Fig 3B). This depth of entrenchment is used as a measure of the degree of sheltering of the vegetation by the topography in the following section.

### Correlation between relief and topography according to forest classification

**Fig 4:**
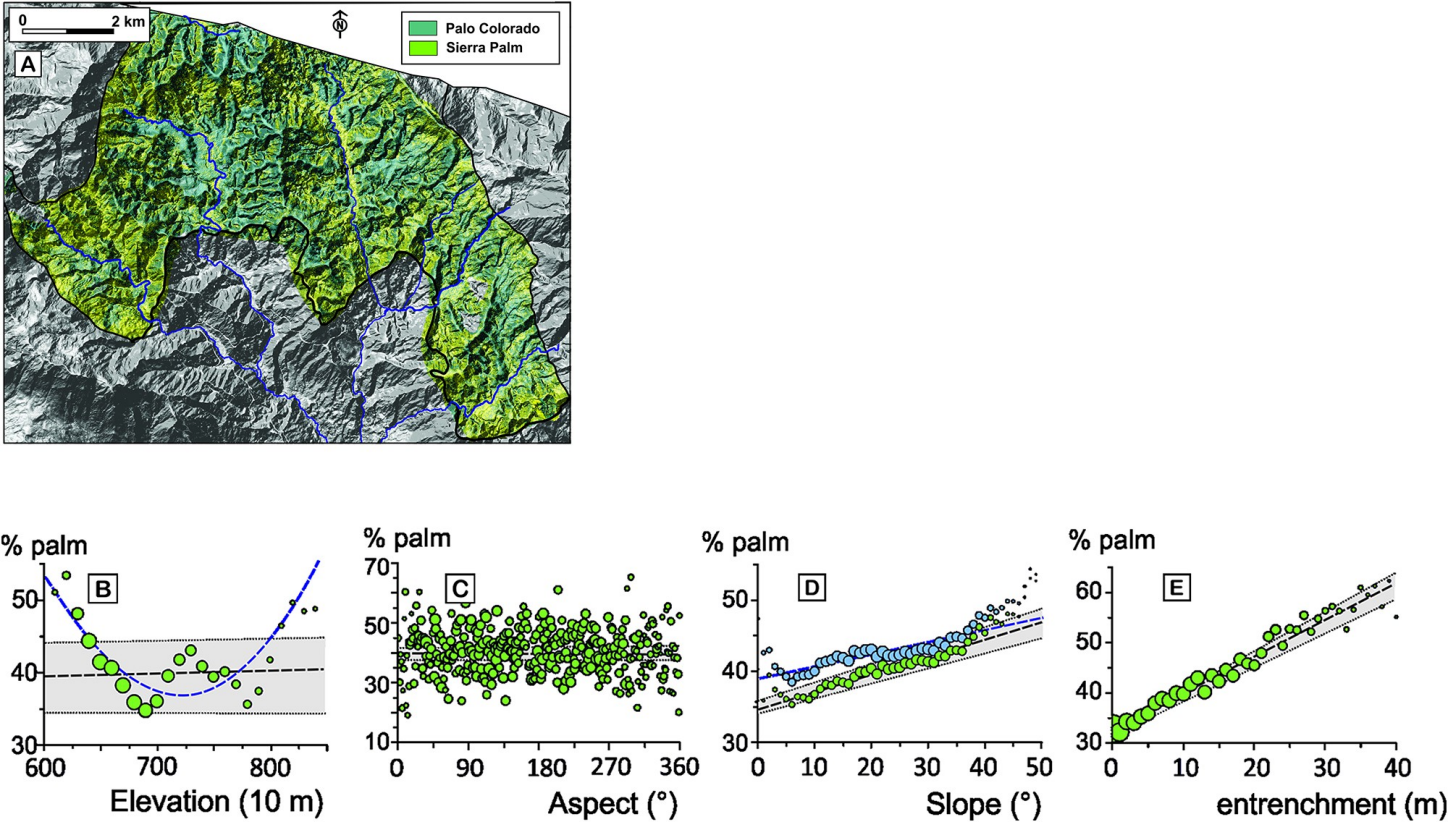
Correlation between topography and forest composition. A: composite classified satellite image of vegetation. Variations of Palm forest, as a function of elevation (B), aspect (C), slope steepness (D), and entrenchment, i.e., sheltering behind hilltops (E) (S2 Table 1 in S2 File). Circle areas are proportional to the areas of forest contributing to each bin of elevation (10m), aspect (1°), slope (1°), and depth (1 m). Dashed lines: linear regressions. Dotted lines bracketing shaded areas: 2σ envelopes. Dashed blue line: second order polynomial regression on elevation (B), and linear regression including floodplains and volcanoclastic aprons (blue circles, D).

Several years of field work on the site, and inspection of satellite images revealed that the distribution of the two main forest types that occupy the study area is controlled by several factors, and among them, topography (Fig 4A). In order to assess this influence, we analyzed how the abundance of Palm forest and of Palo Colorado forest varies as a function of altitude, aspect, slope steepness, and sheltering by surrounding ridgelines (Fig 4B). No linear correlation is observed between aspect or elevation and the percentage of Palm and Colorado forest (Fig 4B and 4C, r^2^< 0.001 and r^2^< 0.03, respectively). A marginally better fit (AIC = 207 vs. 186) is obtained with elevation when using a non-linear relationship, but given that the relationship between rainfall and elevation is linear in the Luquillo Mountains, the use of a complex fitting is not justified. A much stronger correlation is observed for parameters that contrast hilltops and coves, such as slope steepness (Fig 4D, r^2^ = 0.89), or the depth of sheltering behind hilltops (Fig 4E, r^2^ = 0.90). They highlight the propensity of the Palm forest to occupy coves, while the Palo Colorado forest covers hilltops.

### Differences in ^10^Be-drived erosion rates between hilltops and coves

**Fig 5:**
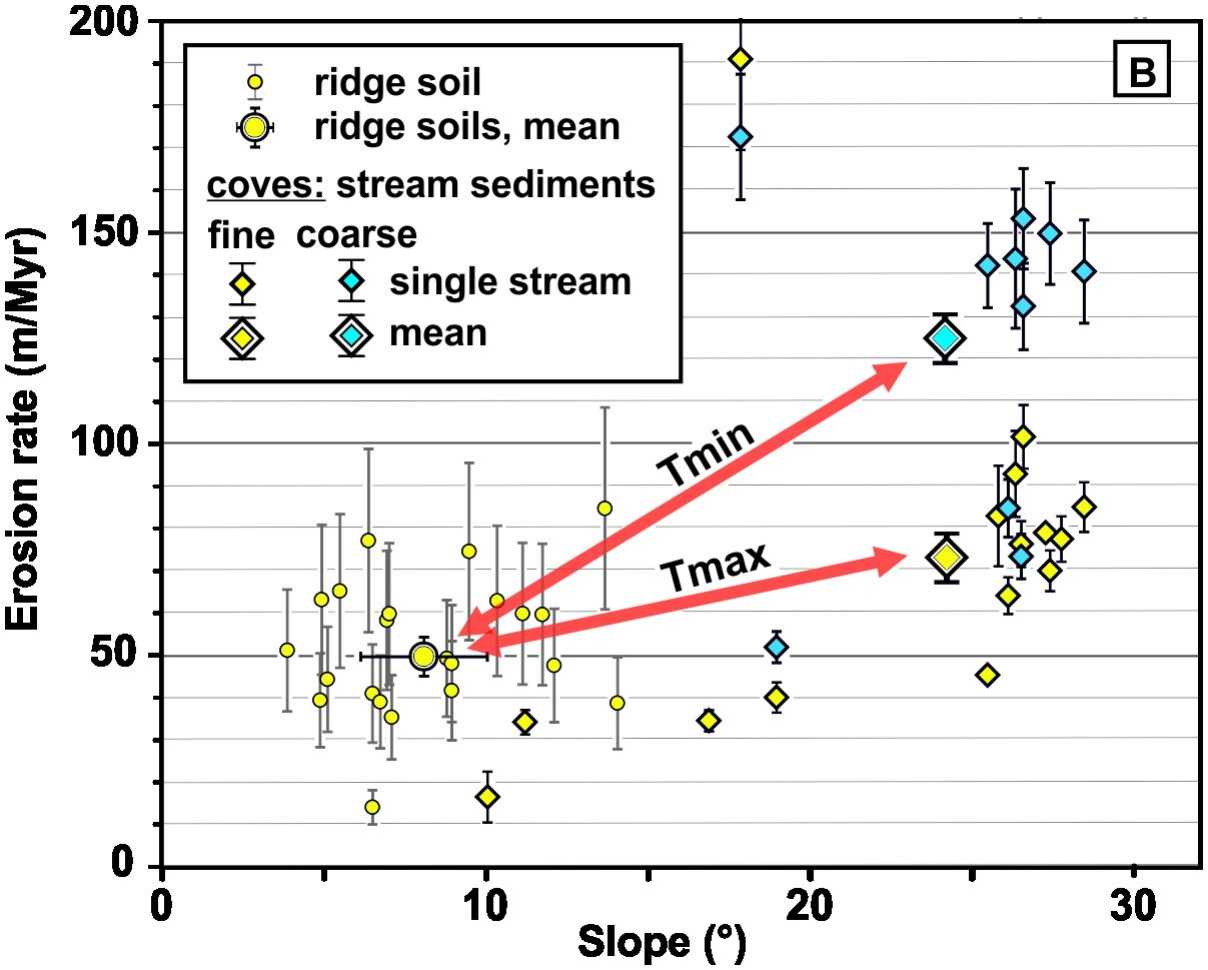
^10^Be-derived denudation rates as function of soil and catchment slope. *Tmin*, *Tmax*: arrows linking average values of erosion rates in the soils to the average values of erosion rates in streams, for the coarse and fine river sediment fractions. These two end-member fractions are used in the calculation of the time required to produce the observed landforms (see discussion).

The erosion of the topsoil on hilltops (S3 Tables 1 and 3 in S3 File) was calculated assuming secular equilibrium between the production of ^10^Be and soil denudation. Secular equilibrium is indeed expected on low-slope, shallow-curvature hilltops where diffusive processes dominate. In the Luquillo Mountains, it has been shown that the prevailing diffusive processes are soil creep, tree throw, and bioturbation [18–20]. We assessed the effect of this assumption on erosion rates by measuring three vertical profiles of ^10^Be concentration with soil depth. The assumption can be tested by fitting theoretical profiles of soil age and erosion rate to the observed concentrations [21, 22]. Two profiles were obtained in broad, low-curvature hilltops (T1OX and TR, S3 Fig 1 in S3 File) They were contrasted to a profile dug into a narrow ridge at the intersection of two coves, where more steady erosion is expected owing to the occurrence of infrequent landslides (PAT, S3 Fig 1 in S3 File). The concentration profile on the narrow ridge clearly documents unsteady erosion, whereas broad hilltop profiles are compatible with steady to near-steady erosion. There, the assumption of steady erosion produces at most a 20% underestimation of the erosion rate. Overall, the obtained erosion rates on the low-curvature hilltops are slow, and highly clustered around 50 ± 14 m∙My^-1^, (n = 19, Fig 5). The tight clustering further supports the assumption of steady exhumation, as an unsteady exhumation would produce more scattering. An earlier published calculation, based on a smaller ^10^Be dataset yielded an hilltop erosion rate of 43 m∙My^-1^ close to our dataset (35 ± 10 m∙My^-1^) if we suppress our added correction for the effect of quartz enrichment in the topsoil [23].

The concentration of ^10^Be in river borne quartz was measured in 25 samples (S3 Table 4 in S3 File) from the bedload of streams that drain various apportionments of broad, shallowly-dipping (< 15°, Fig 3A) low-curvature hilltops and coves. The average slope of the catchments ranges from 10° for catchments that mostly drain low-curvature hilltops, to almost 30° for that mostly drain coves (Fig 5). The erosion rates of hilltop-dominated catchments (15–40 m∙My^-1^) overlap with the lower range of soil erosion rates measured in the broad hilltops (~10–80 m∙My^-1^), possibly reflecting a slight additional accumulation of ^10^Be in the particles between their exhumation on the ridgelines and their arrival into the streams, during the slow, diffusive transport of quartz grains along the broad hilltop slopes. Erosion rates are higher in cove-dominated catchments, up to values of ~140 m∙My^-1^, according to the coarse fraction, and of ~80 m∙My^-1^ according to the fine fraction. These new stream data are consistent with earlier measurements that targeted larger catchments in the study area (in green on Fig 2), that include our smaller catchment. They provide more spatially-averaged erosion rates at 53.0 ± 1.6 m∙My^-1^ according to the fine fraction, and 83.9 ± 6.4 m∙My^-1^, according to the coarse fraction [12]. The coarse fraction-derived erosion rates are more influenced by the coves, because the coarse fragments are produced by decaying corestones unearthed along the sides of coves (S3.3.2.3 in S3 File). By contrast, the finer fraction is produced throughout the landscape by the ubiquitous saprolite, and therefore tends to incorporate more evenly contributions from all areas. By considering the average rates for the coarse (124 ± 38 m∙My^-1^) and fine (73 ± 40 m∙My^-1^) fractions respectively as the end-member minimum rate of cove erosion (because they incorporate a component of hilltop erosion), and the average of hilltop soil erosion rates (50 ± 14 m∙My^-1^), we find that coves erode between >1.5 ± 0.9 and >2.5 ± 1.1. times faster than the hilltop soils, according to the fine fraction and coarse fraction, respectively (arrows, Fig 5). Such a slower erosion of the hilltops was detected using a smaller ^10^Be dataset by Brown, Stallard [9], during their seminal study of ^10^Be erosion rates in the study area. This landscape disequilibrium has also been inferred from an excess in particulate sediments fluxes relative to solute fluxes [24]. Particulate fluxes are generated by surface erosion, while solute fluxes are mostly fed by weathering at the base of the saprolite, several meters to tens of meters below the ground surface [25, 26]. These larger surface fluxes compared to the deep fluxes would imply that the saprolite thins over time. The 45 m-58 m∙My^-1^ deepening rate of the weathering front [26, 27], however, closely matches surface lowering below hilltops. Therefore, this imbalance must occur within the coves. Over time, faster erosion of the saprolite within the coves should bring the ground surface closer to the weathering front. This is indeed what is observed. The corestones, which are residual blocks of incompletely weathered quartz diorite, decrease in abundance from the weathering front upward. They are almost completely absent from the hilltops and unearthed near the floor of the coves, indicating that the floor of the coves lies closer to the weathering front.

## Discussion

### Control on the distribution of vegetation by topography

We interpret the correlation between topographic position (hilltop or cove) and forest type (Palo Colorado or Palm forest) as the result of a control of the distribution of vegetation by topography. This is a common observation, and in tropical areas, such a control exerts itself in a variety of ways that can act in concert [2, 3, 28]. It not the scope of this paper to address precisely their respective contributions in the case at hand. Besides, the atmospheric, biologic, and soil parameters necessary to tease out these contributions would require a variety of measurements conducted in concert. Nonetheless, the wealth of data collected over past decades in the Luquillo Experimental forest provide interesting insights into which characteristics of the topography most likely drive this control. Topography influences precipitation and temperature at the mountain scale in the Luquillo Mountains [11]. The small difference in elevation between hilltops (699 ± 51 m) and coves (692 ± 58 m) in the study area, however, precludes any substantial contribution of altitude-driven variations in temperature and precipitation to the observed pattern. Vegetation experiences differences in insulation, wind shear, and local evapotranspiration between hilltops and valleys that could affect the distribution of forest types. Insulation, however, is only marginally higher on the hilltops (1.7 ± 0.1 MWH∙m^-2^) compared to the coves (1.6 ± 0.2 MWH∙m^-2^), based on the ArcGIS solar insulation function. Insulation, in any case, probably does not affect the distribution of the Sierra Palm, which grows opportunistically from shaded under-canopy to fully exposed brakes [29]. The effects of wind shear on evapotranspiration cannot be appraised in the absence of in situ measurements. However, wind shear likely exerts a influence on vegetation otherwise, through defoliation and breaking during the hurricanes that strike the Luquillo Mountains [30], which affect hilltops more than valleys [31, 32]. Palm and Colorado forests respond differently to hurricanes. Palo Colorado forest tends to experience more branch loss, but rapid regrowth from surviving stems [33], whereas Palm trees tend to snap more, but also reseed rapidly [32].

Topography further influences the distribution of forest types through the routing of surface and near-surface overland flow. Vegetation in the coves grows on steeper slopes (27±9°) than on hilltops (17±8°). Hilltops are therefore not as well drained as coves, and we observed that they commonly support water-logged hypoxic soils. Palm forest grows on well-drained steep terrain [34] but also water-logged sandy floodplains [15]. Likewise, the Palo Colorado forest, while growing here on shallow slopes, grows otherwise on steeper slopes throughout the Luquillo Mountains. Soil dysoxia is therefore not a discriminating parameter. Slope steepness and soil water saturation also affect landslide susceptibility. Hilltops never reach the locally-constrained 30° slope threshold for landsliding [17], whereas landsliding is common in coves (from maps in [16, 31]). Sierra Palm grows as successional vegetation on landslide scars in the Luquillo Mountains [34], and therefore seems to better cope with landsliding than the Palo Colorado forest, and is therefore more adapted to the cove environment.

Topography also influences the distribution of forest types through nutrient routing. In the study area, bedrock weathers to an intensely nutrient-depleted saprolite, and the weathering front lies far below the surface [26, 35]. This strongly limits vegetation access to bedrock-derived nutrients [26, 36]. The forest therefore relies heavily on atmospheric fertilization [37, 38]. It has been proposed that nutrient limitation is the main driver of topographic control on vegetation, in the Luquillo Mountains [39], a suggestion made in other tropical forests [2, 3]. It is characterized by more pronounced nutrient depletion on hilltops than in valleys. The importance of bedrock-derived nutrient limitation at the mountain scale in the Luquillo Mountains is suggested by a positive correlation between surface erosion rates and the delivery of primary minerals to the topsoil, increasing the pool of available cations [12, 36], and by a positive correlation between erosion rates and canopy height [13]. The less intense weathering of the saprolite exposed in the coves compared to the average saprolite [36], and the higher concentration of major solutes in streams draining the coves relative to other landscape domains [40], support the view that the coves receive more bedrock-derived nutrients than the hilltops. Considering the high-impact of these fluxes on vegetation in the area affected by an erosion wave farther down the mountain [13], it is inferred that bedrock-derived nutrient circulation might be the dominant process whereby topography controls the distribution of vegetation in the study area.

### Topographic decoupling between hilltops and coves

If the topography was at equilibrium in the study area, hilltops and coves would be expected to erode at the same rate. Local relief height between hilltops and coves would remain constant, and hillslope steepness would vary progressively along slope, as a function of the divergence in the downward flux of soil with distance from the ridgetop. In the study area, hillslopes abruptly transition across a break-in-slope from shallow-sloping, low-curvature, convex hilltops into steep, highly concave coves (Fig 3A). We interpret the break-in-slope as a line of decoupling between upper and lower slopes, produced by a protracted imbalance in erosion rates, between coves and hilltops, with coves that erode faster than hilltops. The spatial distribution of these variations are similar to the present-day distribution of ^10^Be-derived erosion rates, but would need to exert itself over a period of time much longer than the integration time of the ^10^Be record. Assuming that the topographic envelope used to calculate the sheltering of the forest behind surrounding hilltop represents a first-order approximation of the initial topography from which the current topography has differentiated, and using the present-day differences in ^10^Be erosion rates between coves and hilltops (Fig 5), we find that the present landscape would have started to develop between 0.20 ± 0.08 Ma and 0.44 ± 0.35 Ma, depending of the size fraction of stream sediment considered (S3 Table 5 in S3 File). In their seminal study of ^10^Be erosion rates in the study area, Brown, Stallard [9] conducted a similar calculation. Their calculation was based on a more restricted ^10^Be dataset, and without similar corrections for environmental parameters (such as topographic shielding, vegetation cover, or quartz enrichment). They also started the simulation from an initially flat landscape. They found that topographic differentiation would have started at 1.3 Ma. Suppressing our corrections for environmental parameters we find that, according to their calculations, differentiation would have started between 0.11 ± 0.7 Ma and 3.0 ± 2.7 Ma (S3 Table 5 in S3 File).

The decoupling between hilltops and coves pervades the entire study area and therefore implies that valley deepening is a response to an event affecting the entire area. Valley deepening is most commonly caused by accelerated lowering of the base level of the rivers that drain the valleys. Here, however, the base levels of coves are shallow-gradient alluviated floodplains (Fig 3A) that do not exhibit any indication of stepped, or accelerating base-level lowering. The elevation of the floodplains is in turn controlled, at an elevation of ~ 600 m, by bedrock sills located at the head of prominent river knickpoints that migrate upstream at 1.1–1.5 km∙My^-1^ [10]. The upstream migration of these sills is not expected to impact floodplain lowering farther upstream, unless the floodplains are underlain by a thick erodible substrate that would need to be eroded, in order to keep the floodplains graded to the 600 m elevation of the sills, as the sills migrate upstream. One can calculate what would be the maximum effect the knickpoints on floodplain incision, if the bedrock surface below the floodplains was horizontal upstream of the sills, while the surface slope (1–2%) of the floodplains rises. At the rate of knickpoint retreat (1.1–1.5 km∙My^-1^), floodplains would be lowering at <11-31m∙My^-1^, that is, much more slowly that cove erosion, and even more slowly than hilltop erosion. Besides, it is more likely that the bedrock below the floodplains rises in the upstream direction, and this effect should be even smaller. Last, the rate of floodplain lowering should be unsteady to trigger a drop in base level susceptible to initiate faster incision of the coves, and there is no evidence, for an acceleration of the knickpoints over the past millions of years [10]. Instead the broad, low-curvature ridges between the coves grade gently downslope to the level of the current floodplains (Fig 3B). The coves, therefore, appear to have nucleated inside a shallower landscape, by backwearing into the flanks of the broad hilltops, without associated base level lowering, and the broad hilltops appear to be the remnants of a landscape that was grade to the floodplains, not far above their current elevation (Fig 3B).

Some coves host seeps in their headwaters. Seepage may contribute to cove growth [41], by groundwater-driven piping and landsliding. A dominant contribution of seepage to the conversion of the shallow landscape into a cove-dominated landscape would require an increase in the contribution of groundwater-driven water flow over time, through a deepening of the saprolite, as a result of either an increase in weathering rates at the base of the saprolite, or by a decrease in surface erosion rates. Currently, however, surface erosion rates outpace weathering rates [24]. Most of the water that reaches the forest floor is conveyed as quickflow through macropores in the shallow soil [35, 42]. Little water finds its way into the deeper saprolite, where saturation is only reached during the highest rainfall events [17, 26]. Landslides, away from artificial road cuts, are shallow and translational, removing essentially the soil and topmost saprolite [16]. They do not therefore, in this case, involve deep groundwater contribution. Since most water is routed through the biological soil, we now consider the contribution of vegetation to the formation of the coves.

### Control of topographic development by vegetation

The study area erodes quite slowly (40–60 m∙My^-1^ [9, 12]) considering altogether its average steepness (23 ± 10°, 1σ), the high amount of precipitation is receives annually [11], and the frequent return of hurricanes [43]. The structure of the forest soil has been shown to strongly inhibit slopewash as well as soil saturation during even the highest intensity rainfall events [18], owing to an efficient routing of water through connected macropores in the upper soil [42], where porosity reaches 75%, decreasing to 45% at 0.5 m depth [26]. It appears, therefore that forest soil structure dampens soil erosion in the Luquillo Mountains [10]. Given that the Palo Colorado and Palm forests have strikingly different above-ground and soil structures [44], one can expect that these differences will variably affect slopewash, quickflow, soil saturation, and landsliding. The Palm forest is dominated by mature, monospecific stands of Sierra Palm, under which little undercanopy vegetation grows [34]. We observed that the mineral soil, exposed between jams of palm leaves accumulated under the action of slopewash, bears the marks of rain splash and scattered lags of quartz sand. By contrast, the Palo Colorado forest hosts a much denser undercanopy that prevents rain splash and direct dripping from the canopy. The mineral soil is protected by a continuous litter, which itself overlies thick organic root mats, through which most runoff is conveyed as quickflow in large macropores, above the clayey mineral soil. Erosion by overland flow therefore appears more intense under Palm forest than under Palo Colorado forest. Hillslope steepness being equal, landslides may occur more frequently under Palm forest, because Palm roots are less entangled than in the Palo Colorado forest, probably offering less resistance to slope failure. In addition, Palm trees are also more frequently destroyed by hurricanes, leaving the ground even less protected. In contrast, in the Palo Colorado forest, trees more commonly survive branch loss, and are rarely uprooted [30].

**Fig 6:**
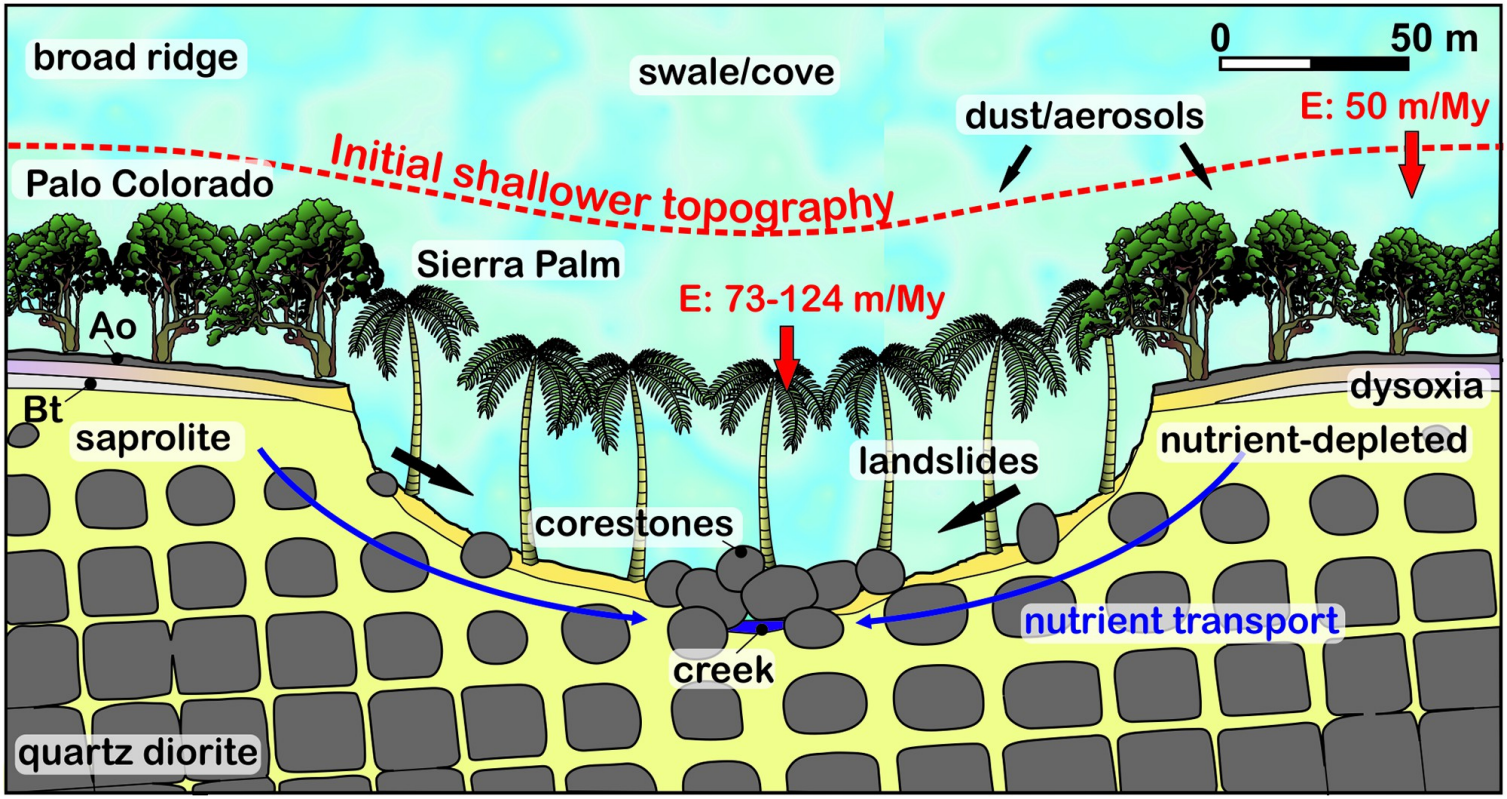
Conceptual model of relief development due to faster erosion in the coves. E: denudation rate from terrestrial ^10^Be analysis. Ao: soil litter, Bt: clay accumulation horizon (see S2 Fig 2 in S2 File for corresponding field photographs).

We propose that, at the start of the process of topographic differentiation, Palm trees were slightly more abundant in the valleys, promoting faster erosion of the soil on slopes that were not initially distinctively steeper than on ridgetops (Fig 6). The progressive deepening of the valleys reinforced the segregation between Palm and Colorado forests according to topography, in turn promoting a sharpening of the difference in erosion rates between hilltops and coves, through a positive feedback. The coves would end up being sites where steep slopes are concentrated, which in turn favors the occurrence of landsliding, soil saturation, and ground sapping. Another positive feedback would then develop, as the Palm trees, apart from being able to form mature forest, are also part of the successional vegetation on landslides. These processes would act such as to further enhance to topographic segregation of vegetation and topographic differentiation.

If topographic differentiation is thus originally and primarily driven by topographically-controlled vegetation, then topographic differentiation can be expected to have started when the study area, currently located at elevations of 600–750 m, was settled by Palm and Palo Colorado forest. Our simple calculation, which assumes a linear development, suggests that such an event would have taken place between 0.11 ± 0.03 and 1.4 ± 0.3 Ma. This timing is consistent with the age of the Luquillo mountains, which started to rise < 4.5 My ago [10]. Palm and Colorado forests probably initially settled the highest peaks of the Luquillo Mountains, before spreading downslope as the mountains continued to rise, and as the mountains further collected more precipitation. Settling of the study area would have started when the study area became high enough to support these montane forests, which currently grow above 550 m [14]. Settling could have occurred later, following a so-far undocumented substantial change in the Caribbean climate. Clade analysis of endemic mountain frogs independently suggests that the biodiversification of mountain coqui frogs in the Luquillo Mountains occurred between 180 ka and 1.5 Ma [45]; biodiversification appears to have started when the Luquillo Mountains became a mountain high enough to support the forest types that host these frogs, isolated from the main mountain range (Cordillera Central). This independent dataset suggests that the differentiation of topography, the reinforcement of topographic control on vegetation, and the biodiversification of the forest could be rooted in the same event, and that landforms, vegetation and animal species have coevolved ever since.

At the scale of the Luquillo Mountains as a whole, the preponderant role played by vegetation in landscape evolution is highlighted by the fact that the forest has protected the mountains from rapid hillslope erosion and stream incision, over the past millions of years [10]. The forest has thereby prevented a fast topographic decay of the mountains, ensuring the very persistence of the montane forest habitat. The morphological and geological simplicity of the studied part of the forest reveal local-scale trends that appear to reflect at greater spatial resolution some aspects of the coupling between vegetation and topography which are otherwise blurred by other parameters in other parts of the Luquillo Mountains characterized by the greater lithological complexity and more complex geomorphological evolution. This is the case in most landscapes, and for this reason, the contribution of vegetation to the development of mesotopography is largely overlooked. Yet, this frontier area of research may prove an important component of landscape evolution that should be taken into account when interpreting and modeling natural landscapes.

## Conclusions

A strong correlation between topography and vegetation is observed at the scale of hilltops and valleys, within a slowly eroding part of the Luquillo Mountains. Hilltops tend to attract Palo Colorado forest, while the surrounding, deeply concave valleys (coves) tend to host Palm forest.

Topography can control the distribution of these forest types in various ways. In landscapes strongly depleted in bedrock nutrients, such as here, fluxes of bedrock nutrients likely play a significant role in topographically-driven forest differentiation.

An inventory of the concentration of cosmogenic ^10^Be produced in quartz located in hilltop soils and stream sediments reveals that the coves are eroding faster than the hilltops, confirming, and expanding earlier observations.

A systematic break-in-slope separates the shallow-dipping, low-curvature hilltops, from the surrounding, deeply concave coves. This topographic signature can be produced by perpetuation of the current imbalance in erosion between hilltops and coves over 0.1–1.5 My.

We interpret the differences in erosion rates between hilltops and coves as driven initially by the difference in ground protection offered by sharp differences in subaerial and underground structure provided by the canopy-forming trees, their undercanopy, the litter, and the root mats between the two types of forests. Palo Colorado forest more efficiently suppresses soil saturation, slope wash, and landsliding than the Palm forest.

Because these forest types are controlled by topography, a positive feedback exists that deepens the coves with respect to the hilltops over time, concentrates Palm forest in valleys and Palo Colorado forest on hilltops. The perpetuation of the process is ensured by the ability of the Palm forest to persist once the coves become steep, deeply confined between ridges, and struck by landslides.

The initiation of the process may correspond to the time at which the study area was settled by the Palo Colorado and Palm forests. It also corresponds to the time at which local frog species, hosted by these forests, also started undergoing biodiversification.

## Supporting information

S1 File LiDAR data processing. (PDF)

S2 File Forest classification and ground cover. (PDF)

S3 File In situ-produced terrestrial cosmogenic ^10^Be data. (PDF)
