# Supplementary material for: Shaping of topography by topographically-controlled vegetation in tropical montane rainforest
Source: PLoS One. 2023 Mar 9;18(3):e0281835. doi: 10.1371/journal.pone.0281835 (PMC9997930; doi:10.1371/journal.pone.0281835)
Supplement: S1 File — (PDF) [file pone.0281835.s001.pdf]

Supporting Information

S1. LiDAR data processing

Airborne LiDAR data were collected by the National Center for Airborne Laser Mapping (NCALM) with an Optech GEMINI ALTM (Telodyne Optech, Ontario, Canada) and an Applanix POS/AV 510 OEM with embedded BD950 12 channel 10 Hz GPS receiver (Applanix Corp., Ontario, Canada) on board a Cessna Skymaster (Cessna, Witchita, KS). The data used here acquired in May 2011. The laser wavelength was 1,047 nm, laser pulse frequency was set to 100 Hz, beam divergence was 0.25 mrad (1/e), scan frequency was set to 55 Hz, scan angle was set to ±15°, and scan cut-off was ±2°. Flights were conducted at a speed of 60 m/s. The width of the swaths was 277 m, with 50% overlap between swaths. Point density was approximately 14 points m^{-2}. Discrete returns were horizontally referenced to NAD 1983 UTM Zone 20 N (EPSG: 26920) and vertically referenced to NADV 1988 (EPSG: 5703). Data are available online through OpenTopography (www.opentopography.org, **doi**: https://doi.org/10.5069/G9BZ63ZR).

Following standard procedures, the discrete laser returns were rasterized at a spatial resolution of 1 m^2. The lowest z-values, provided by the last returns were classified as ground points and used to construct the DEM [1]. Persistent cloudiness and dense vegetation resulted in a relatively low density of ground points [1]. The low density of ground points makes it difficult to discriminate dense undercanopy from corestones and other high-roughness terrain, generating noise in the 1 m-resolution data. To overcome this issue, we resampled the 1 m-resolution LiDAR DEM at 5 m-resolution to remove noise from the original DEM. The 5m-resolution DEM (S1 Grid 1) was used to calculate elevation, slope aspect, slope steepness, and depth of sheltering behind the hilltops. This later variable was obtained by subtracting the 5m-resolution DEM from a surface envelope (S1 Grid 2 and contour lines on Fig. 3B) passed through the hilltop crests), elevations, extracted from the 5 m-resolution DEM, from successive broad hilltop patches, and by cubic spleen interpolation between these patches.

S1-Grid 1. 5m- DEM of processed point cloud data.

S1-Grid 2. Surface envelope pinned to current broad ridges.

1. Wolf JB, G.; Willenbring, J.; Porder, S.; Uriarte, M. Abrupt Change in Forest Height along a Tropical Elevation Gradient Detected Using Airborne Lidar. Remote Sensing. 2016;8:864.
