# Supplementary material for: Shaping of topography by topographically-controlled vegetation in tropical montane rainforest
Source: PLoS One. 2023 Mar 9;18(3):e0281835. doi: 10.1371/journal.pone.0281835 (PMC9997930; doi:10.1371/journal.pone.0281835)
Supplement: S2 File — (PDF) [file pone.0281835.s002.pdf]

S2. Forest classification and ground cover

Persistent cloudiness over the El Yunque National Forest makes it difficult to obtain cloudless image during the acquisition of single satellite scenes. A classified mosaic of the forest (Fig 4A) was obtained by combining the visible bands of partially cloudy multispectral images, namely: GeoEye 1© 2010 Maxar, 05th and 13th December 2010, Ikonos 1© 2010 Maxar, 1st September 2010, and Worldview 1© 2007 Maxar, January 8th 2007, projected to GCS_WGS 1984, provided through the Polar Geospatial Center (University of Minnesota—Twin Cities, https://www.pgc.umn.edu). Oriented classification was conducted using ArcGIS 9. The forest was classified into the one of the two types of forest that occupy the mountain in the area of investigation, namely the Sierra Palm forest and Palo Colorado forest (Luquillo Long-term ecological research, El Yunque Forest Assessment Plan 2014; Puerto Rico GAP project, https://data.fs.usda.gov/geodata/other_fs/IITF/index.php). The selection of the training samples and the post-processing evaluation were conducted visually using 40 cm to 60 cm-resolution panchromatic images corresponding the multispectral images here above . Results were ground-proofed (S2 Fig 1) along mostly off trail transects in areas were open views allow ground observers to see the canopy.

S2- Grid 1. Georeferenced forest classification in the area of analysis of the relationship between forest type and topography (GeoTiff)

S2- Shapefile 1. Border of the area of investigation (shapefile .shp)

S2- Shapefile 2. Border of the area of analysis of the relationship between forest type and topography (shapefile. shp)

S2-Shapefile 3. Ground-proofing tracks.

S2-Table 1. Forest composition-topographic parameter correlations

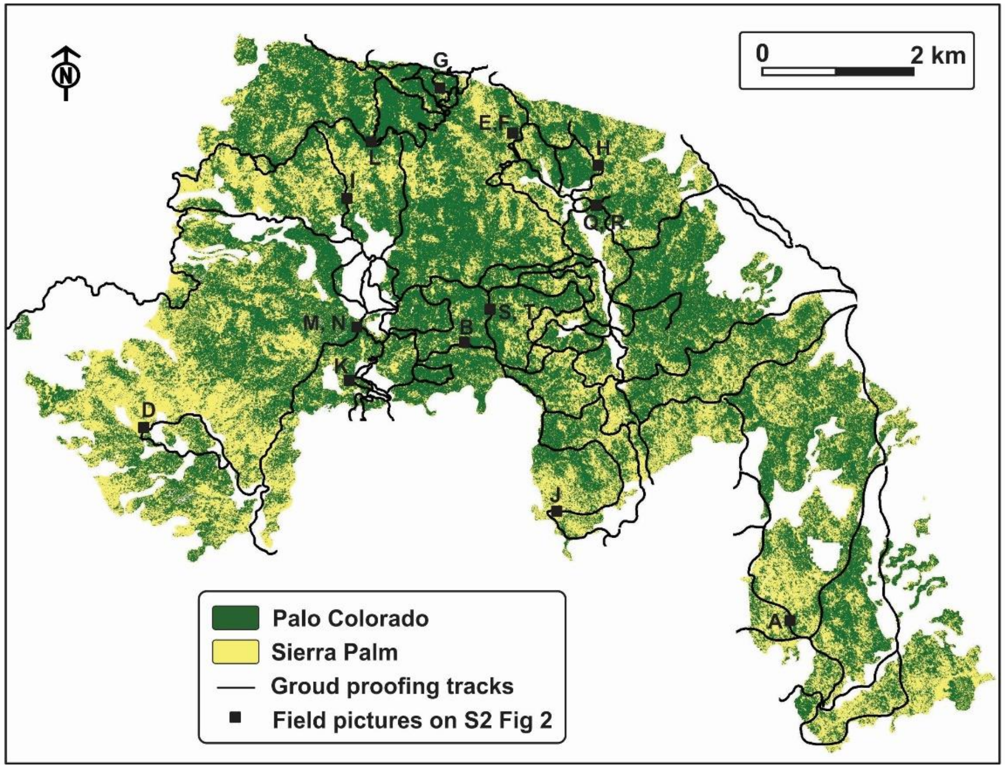

S2 - Figure 1. Map of the classified forest showing ground-proofing tracks and location of field pictures of S2 Fig 2.

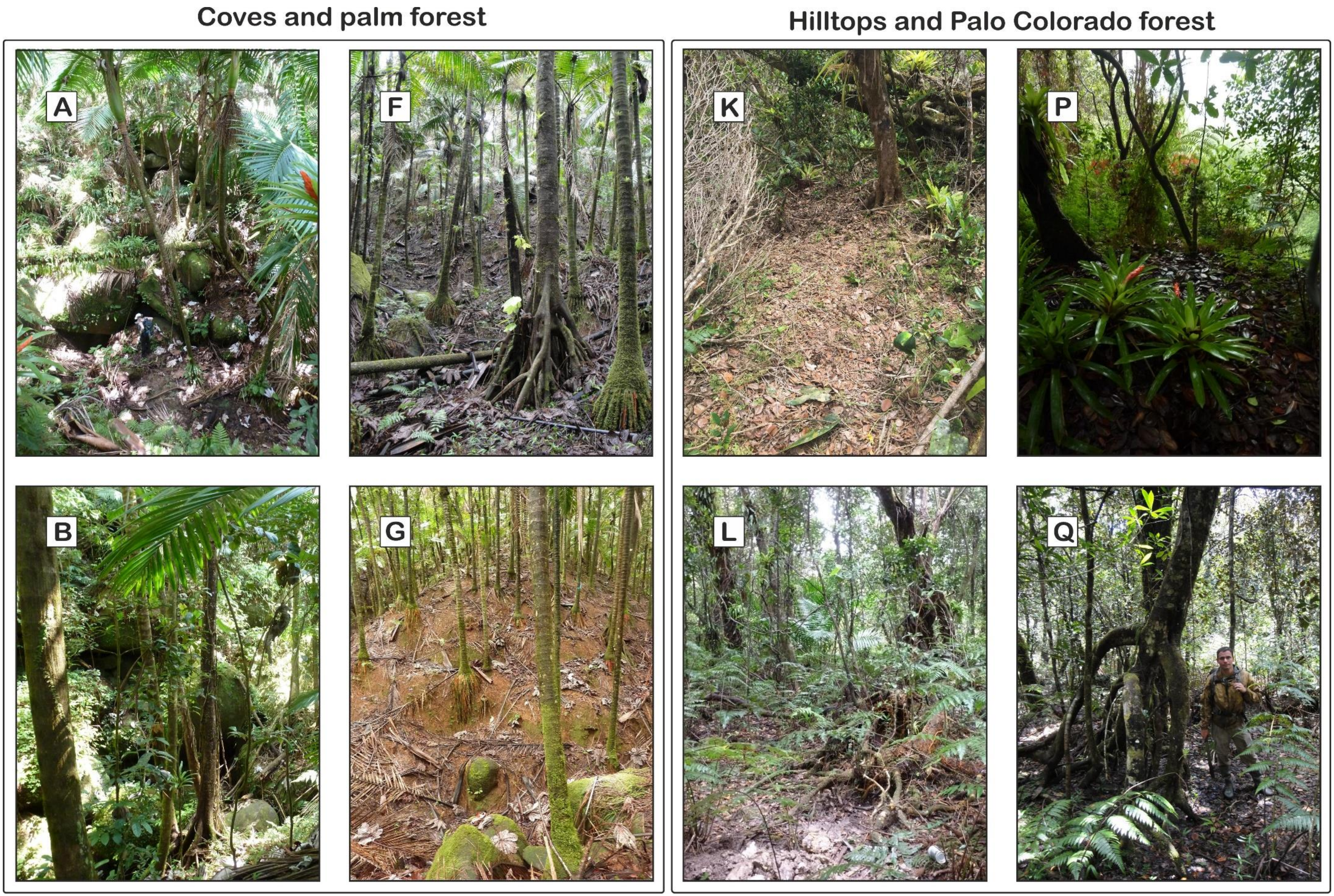
Coves and palm forest
Hilltops and Palo Colorado forest
A
F
B
G
K
P
L
Q

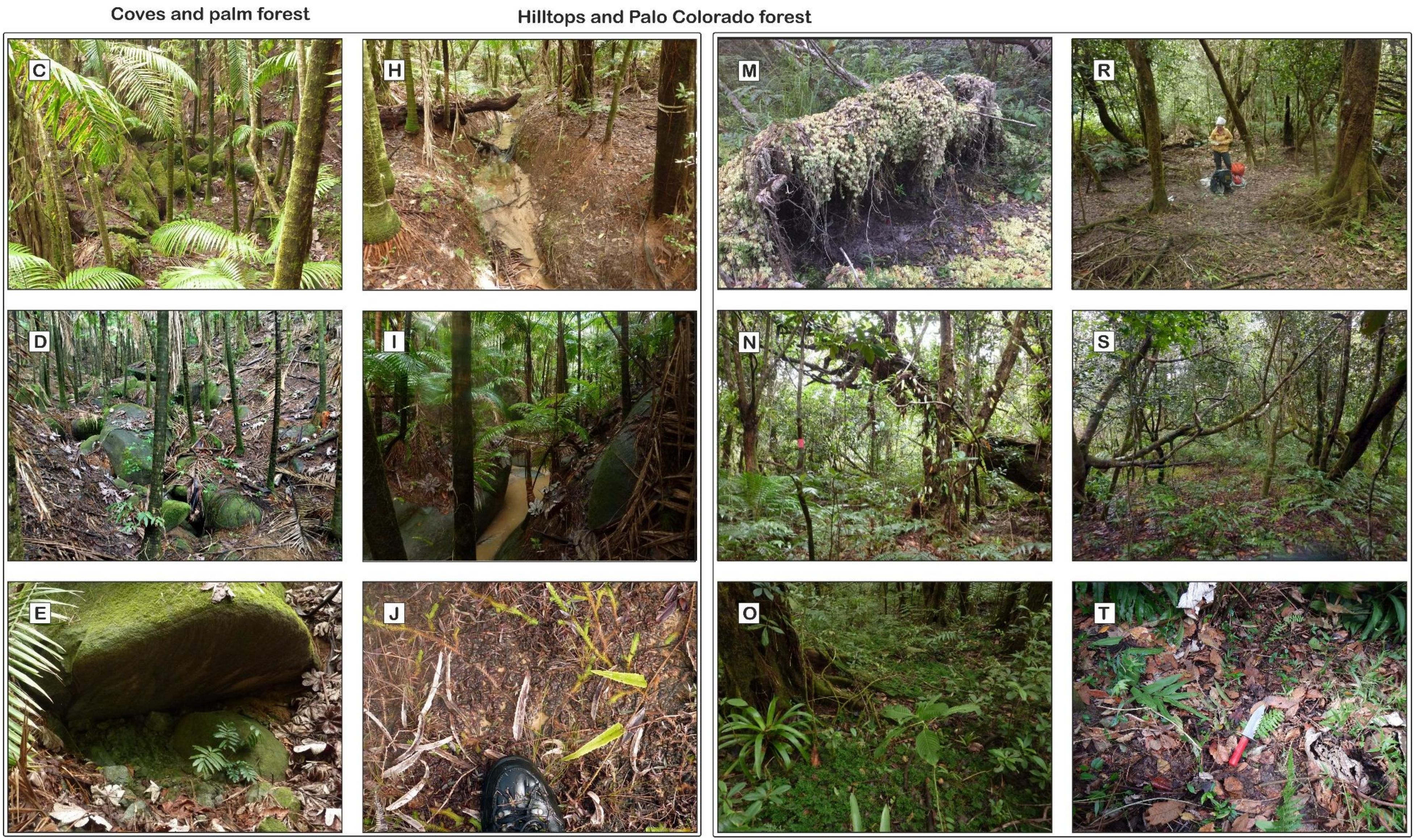

S2 - Figure 2. Field pictures of the Palo Colorado and Palm forests, in the area of investigation (see S2 Fig. 1 for location).

A-E: corestone accumulations in palm coves. A and B: large corestone piles (note person for scale on B), C and D: smaller accumulations above creek beds, E: disintegrating corestone feeding the coarse fraction of fluvial sediments.

F-J: palm forest in coves, upslope or downstream of corestone piles. F: details of palm root systems, G: litter-devoid soil exposed under palm trees, H: litter-devoid soil under palm trees near sandy creek, I: typical mixed landscape of palm trees, corestones and creek in a cove, J: lag of coarse quartz sand on the ground surface, partially covered by algi and lycopods.

K-T: Palo Colorado forest. K: leaf-covered root mat at a site investigated for shallow geophysical characterization of litter thickness, L: anoxic A horizon below litter, M: partially uprooted root mat showing the shallow root anchorage, underlying dysoxic soils, and sphagnum and litter ground cover, N: toppled, but unbroken tree, with vertical regrowth, O and P: typical ground coverage of selaginella, bromeliads, ferns, root mats and leaves, Q: stilted roots above ground litter, R-T: large, flat hilltops with mature Palo Colorado forest and extensive leaf litter above root mats and gray, disoxic soil above deeper, bright yellow-orange oxic pedons (not shown).

Photo credits: Jane Willenbring (F, Q) and Gilles Brocard (all other photographs).
