# Supplementary material for: Shaping of topography by topographically-controlled vegetation in tropical montane rainforest
Source: PLoS One. 2023 Mar 9;18(3):e0281835. doi: 10.1371/journal.pone.0281835 (PMC9997930; doi:10.1371/journal.pone.0281835)
Supplement: S3 File — (PDF) [file pone.0281835.s003.pdf]

S3. In situ-produced terrestrial cosmogenic ^{10}Be data

S3.1: Sampling strategy and protocol

S3.1.1. Stream sampling

Coves (valleys) in the study area are frequently struck by shallow landslides and their thalweg are chocked by piles of corestones. These characteristics generate a sharp spatial variability in surface ^{10}Be concentration, which is overcome through spatial averaging. Ground obstacles hinder the use of the soil sample amalgamation technique, based either on a grid regular-spaced sampling points, or truly random sampling. Instead, we use the natural averaging effect of stream catchments by measuring ^{10}Be in quartz transported by streams draining the study area. Stream sediments were collected on sand-and-gravel bars at various locations, far enough from stream junctions to ensure thorough mixing of the bedload.

The ^{10}Be contained in the riverborne quartz is produced by cosmic rays within the uppermost few meters below the ground surface. The residence time of quartz grains with the depth range over which ^{10}Be is produced depends on the rate at which soil is removed by erosion. At steady state, the concentration of ^{10}Be at the ground surface is controlled by its erosion rate, and the concentration of ^{10}Be in river-borne quartz sand is then the average of hillslope erosion among the parts of the catchment feeding the stream with quartz. The catchments studied here are underlain by three rock units: the Río Blanco quartz diorite, low-grade metamorphic basaltic to andesitic volcanoclastic country rocks, and an aureole of hornfelsed volcanoclastics around the quartz diorite intrusion. Volcanic rocks and hornfels contain microcrystalline metamorphic quartz, but upon application of the standard quartz extraction protocol [1], grains are decomposed into very small particles (< 65 µm) and do not contribute to the fractions used for subsequent measurements. Another source of quartz is provided by Saharan dust, which is abundantly deposited across the catchment [2]. Its small size (<20 µm [3]) ensures it does not contribute to the measurements. Quartz diorite is therefore the only substrate contributing quartz. The dataset consists of 15 streams draining the sub-catchments of five larger, previously measured catchments [4, 5].

S3.1.2. Soil sampling

Hilltops within the study area are broad, shallowly convex, devoid of landslide scars. The A horizon is well mixed by bioturbation and tree uprooting, which drive particle diffusion across the topsoil. The residence time of quartz within the ^{10}Be production zone (a few 1,000s to 10,000s years) is much larger than the average tree life expectancy (a few 10s to a few 100s of years). As a result, on hilltops, ^{10}Be concentration is spatially averaged by soil diffusion, and the ^{10}Be concentration of grab surface soil samples is regarded as representative of the concentration over several square meters of ground surface. The soil dataset consists of 27 samples of topsoil (0-10 cm-depth) taken at widely distributed hilltop locations (Fig 2 and S3 Table 3). The dataset also includes 16 samples taken to construct 3 depth-concentration profiles (Fig 1 and S3 Table 1). Two of these profiles were dug into broad ridgetops, and one into a narrow hilltop which separates two adjacent coves headwalls. These profiles are aimed at testing the steadiness of ^{10}Be accumulation in the studied soils.

S3.2. Quartz isolation, ^{10}Be extraction and measurement

Soils and stream sediments were sieved into phi-scale size fractions. Clasts from fractions >500 µm were crushed and sieved to retrieve the 250-500 µm fraction. Quartz isolation, purification, and dissolution, ion exchange extraction and precipitation of beryllium were performed at the University of Pennsylvania Cosmogenic Isotope Laboratory (PennCIL). A ^{9}Be carrier (Scharlau BE03450100) with a measured ^{10}Be/^{9}Be ratio of $1.5 \cdot 10^{-15}$ was added to each sample. Beryllium hydroxide was precipitated at pH 8-9, oxidized to BeO over an open butane-propane flame, and mixed with Nb powder in steal targets for AMS measurement. The ^{10}Be/^{9}Be ratio of the samples and procedural blanks was measured by accelerator mass spectrometry (AMS) at PRIME lab, Purdue University. Results were normalized to the 07KNSTD standard, with an assumed ^{10}Be/^{9}Be ratio of $2.79 \cdot 10^{-11}$. The average ^{10}Be/^{9}Be ratio of the procedural blanks is $4.3 \pm 0.3 \cdot 10^{-15}$ (n=7, 1σ). Reported one-sigma uncertainties (S3 Tables 1 and 4) encompass uncertainties on Purdue AMS measurement, primary standard, blank corrections.

S3.3. Conversion of ^{10}Be concentration into denudation rates

S3.3.1. Soils and rock surfaces

The calculation of surface erosion rates based on grab surface samples assumes that surface erosion is regular enough to be regarded as steady over the duration of exposure of ground particles to cosmic rays [4]. The shallow slopes of the hilltops are not conducive to slope failure, and no slope scars are discernible on them. However, the trace of very infrequent (with a return period of thousands of years), shallow landslides could be effaced by diffusive processes and would challenge this assumption. We verified whether the assumption is valid by measuring the evolution of in-situ produced ^{10}Be concentration in quartz with depth along three vertical profiles (S3 Table 1). Two profiles document the decrease of ^{10}Be with depth within two broad, shallow-sloping, low-curvature hilltops (T1OX and TR, S3 Fig 1A). A third profile documents the decrease of ^{10}Be with depth within a narrow ridge, located at the intersection of two steep cove headwalls, affected by landslides (PAT, S3 Fig 1A). All three profiles display the expected overall increase in ^{10}Be concentration toward the surface, which plateaus across oxic A and B pedons that form the upper part of these profiles. Bioturbation does not affect surface concentration at steady state, but increases both time required to reach steady state and affects the depth distribution of ^{10}Be concentration [4]. Bioturbation within profiles TR and PAT homogenizes ^{10}Be concentration in the upper soil. By contrast, concentration also decreases across the topmost dysoxic pedon in T1OX, probably because dysoxia lowers the levels of biological activity and reduces mixing.

Ideal depth-concentration profiles that assume steady upward displacement and surface erosion were fitted to the measured profiles [6]. The modelling assumes progressive build-up from never-exposed material, until secular equilibrium is reached, and models ^{10}Be concentration with depth as a function of exposure age and constant surface erosion rate. Various combinations of exposure age and erosion rates are selected by Monte Carlo sampling were compared to the data. A population of best-fit profiles is then used to define upper and lower possible values for erosion rates and ages. Best-fit values in the case of the very broad and flat hilltop T1OX all lie within the range of steady-state conditions (S3 Fig 1B). They provide well-constrained, narrow ranges of possible erosion rate, and an infinite range of ages. In contrast, values in narrow hilltop PAT imply recent exposure (<40 ka) and much lower ^{10}Be concentrations. This distribution implies unsteady erosion, with some infrequent stripping events, most likely produced by landsides. This is compatible with the morphology of the hilltop, which steeply sloping sides are susceptible to landslides during high rainfall events. Low-curvature hilltop TR displays an intermediate distribution of age-erosion rate couples. Because its soil is more oxic than the soil of T1OX, these difference represents the combined effects of bioturbation on the deviation from an undisturbed depth-concentration profile, with a

possible component of infrequent, accelerated stripping, or/and a return to equilibrium slowed by bioturbation. If TR actually erodes at the same rate as T1OX, then the assumption of steady-state in TR generates a ~20 % overestimation of its actual erosion rate. The high clustering of erosion rates among low-curvature hilltops (Fig 5 and S3 Table 3: all hilltops except narrow hilltops PAT-xx) suggests, first, that these hilltops tend to erode at the same rate, and, second, that disturbances do not generate enough unsteadiness in the ridges to challenge the steady-state assumption.

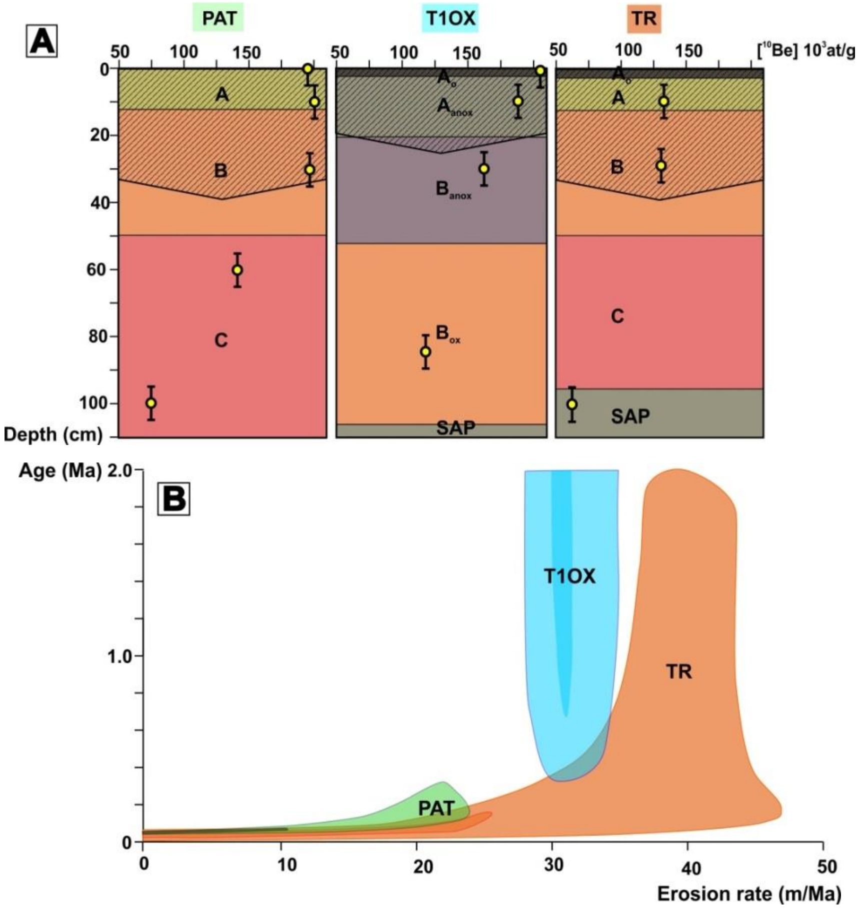

S3 - Figure 1. Evolution of ^{10}Be concentration with depth along tree soil profiles. [A] Soil profiles from broad hilltops (T1OX and TR) and from one narrow ridge at the intersection of two coves (PAT). A_o: litter, A: eluvial pedon, B: illuvial pedon. C: partially destructured saprolite, SAP: saprolite, Anox: anoxic, hatched: visibly bioturbated (traces of worm burrowing). [B] Best-fitting values of erosion rate and soil age to the measured ^{10}Be concentration profiles, using the modelling technique of Hidy, Gosse [6] for the three profiles. Shaded areas indicate acceptable fit, darker shaded areas indicate best fit regions.

Sample name	Easting	Northing	Elevation	Quartz used	[^{10}Be]
	(°)	*(°)*	*(m)*	*(g)*	*(10^4 at g^{-1})*
PAT-SS<br>PAT-10	-65.7950	18.2934	750	17.3<br>60.7	19.0 ± 0.7<br>20.1 ± 0.5

PAT-30				53.9	19.2 ± 0.5
PAT-60				60.8	14.4 ± 0.3
PAT-100				49.3	7.6 ± 0.3
T1OX1-SS				50.1	20.9 ± 0.5
T1OX1-10	-65.7866	18.2856	650	56.3	19.1 ± 0.4
T1OX1-30				45.7	16.5 ± 0.4
T1OX1-85				58.8	11.9 ± 0.3
TR-10				41.3	13.1 ± 0.5
TR-40	-65.7947	18.2913	802	45.9	12.7 ± 0.5
TR-100				44.1	6.3 ± 0.4

S3 - Table 1. ^{10}Be profiles locations and sample concentrations for the data used to assess the steadiness of denudation on hilltops.

S.3.3.2. Stream sediment

S3.3.2.1. *Catchment averaging*

Catchment-wide terrestrial ^{10}Be erosion rates rely on a series of assumptions that the studied area favorably meets. For this very reason, it was chosen to ground-truth the method in some of the earliest tests of this approach [4, 7]. One requirement is that quartz concentration is homogeneous throughout the feeding catchment, and that quartz is contributed equally by all parts of the landscape, such that no weighting for spatial variations in quartz concentration needs to be applied. Quartz distribution in the substrate composed of quartz diorite is homogeneous at spatial scales of 10^1 to 10^2 meters. Its quartz content is typically 21-25 % [8] and shows little variation, except near the batholith border. Areas underlain by quartz diorite amount to 52-100 % of the sampled catchments. They are the only providers of quartz in the analyzed fractions. Catchment-wide calculations are therefore restricted to the areas underlain by quartz diorite.

S3.3.2.2. *Topographic and vegetation shielding*

The LiDAR DEM (S1) was used to calculate topographic shielding. Persistent cloudiness, thick under-canopy, and rough terrane hindered acquisition, such that the resulting 1-m-DEM fails to accurately reproduce meter-scale details of the topography, especially along corestone-strewn corridors (S2 Figs). Although the highest precision DEM is desirable for calculating topographic shielding, the 1m-resolution DEM was resampled at five meters to remove the most prominent artifacts. Holes resulting from gaps in LiDAR acquisition cover 3% of the study area. The resulting holes in 5m-resolution DEM were patched with the 10 m-resolution USGS National Elevation Dataset (United States Geological Survey (2021). United States Geological Survey 3D Elevation Program 1/3 arc-second Digital Elevation Model. Distributed by OpenTopography. https://doi.org/10.5069/G98K778D). Catchment-averaged topographic shielding reaches 5-9% over the 5-m-LiDAR and less than 2% over the 10-m-DEM. To correct for smoothing over the 10-m-DEM, a 6% shielding value was applied to the patched areas.

Two approaches where then used to calculate catchment-integrated production rates. In the first one, the CRONUS online calculator (https://hess.ess.washington.edu/) was used to calculate ^{10}Be production as a function elevation across the study area. A grid of production rates was produced using this elevation function. The shielding grid and the elevation grid where combined to calculate catchment-integrated production rates. Since the CRONUS calculator does not use production rate as an input, we iteratively adjusted the values of catchment-averaged elevations

provided to the calculator such as for the calculator to generate a production rate equal to our calculated basin-integrated production rate. In the second approach, basin-averaged elevation and shielding values were entered in CRONUS to calculate erosion rates. The two approaches provide very similar estimates, as the resulting catchment-averaged erosion rates diverge by less than 2%, owing to the modest amount of topographic shielding and limited elevation range of the quartz-contributing areas.

S3.3.2.3. *Choice of the sediment fraction*

Brown, Stallard [4] showed that ^{10}Be concentration decreases systematically with increasing grain size, through each one-phi size increment, from -3 to 4 (8 mm to 0.063 mm) in the bedload of two streams draining the studied area. This dependency is a solid feature that characterizes the bedload of all streams draining the Río Blanco quartz diorite stock [5]. Therefore, in order to fully catch the spread of ^{10}Be concentration across fractions, we analyzed at each site a fine sand (0.63-0.125 mm) and the coarsest available gravel fraction (≥ 4 mm), where available (S3 Table 4).

This dependency is thought to reflect the upward comminution of bedrock fragment within the upper few meters of the soil profile [4]. This model is not the most suitable in the study area, however, because the bedrock weathers directly, across a very narrow weathering front, into a sandy saprolite and meter-sized corestones [9, 10] that are not mobile in the streams. Besides, the saprolite is more than a few meters thick, and sometimes several tens of meters thick [11, 12], such that the most of the comminution takes place below the ^{10}Be production zone. We therefore use an alternate model to explain the grain-size dependencies, which relies on the uneven geographic distribution of the corestones [5]. In this model, the saprolite releases individual quartz crystals with a maximum size of 2 mm across the landscape. The corestones are only unearthed in the coves, were they constitute the only source of quartz-bearing sediment grains >2 mm. This difference in sediment provenance introduces systematic biases in the calculation of ^{10}Be-derived erosion rates in various ways. First, the coarse fraction is exhumed in coves, where it is preferentially shielded from cosmic rays by the surrounding hilltops, compared to the sand released by the saprolite, which tracks from all landscape positions. Second, the attenuation of cosmic rays is faster in the corestones, which have a density of 2.7 g cm^{-3} in corestones, than in the saprolite and soils which have an average wet density of 1.6± 0.2 g cm^{-3} at field capacity [13]. Third, quartz concentrates in the upper soil horizons [11, 14] owing to the preferential entrainment and dissolution of clay minerals. This effect does not apply to corestones.

A sensitivity analysis (S3 Table 2 and Fig. 2) shows the successive contributions of these parameters to the observed differences in apparent catchment-averaged erosion rates between the coarsest and the finest fraction. Erosion rates are calculated assuming an average soil density of 2.1 g cm^{-3} (model NC). Then an 8% reduction in production rate, which is the average of the topographic shielding from cosmic rays in the coves, is applied to the coarse fraction, while catchment-specific shielding values are applied to the fine fraction (model TC). A 7% vegetation shielding is then added (model TC-VC). The following step explores the effect of variations in ground density, ascribing a soil density of 2.7 g cm^{-3} to the coarse fraction (corestones) and 1.6 g cm^{-3} to the fine fraction (soils, model TC-VC-DV). The effect of quartz enrichment is then introduced (model TC-VC-DV-QE), followed by time-dependent variations in ^{10}Be production rate, such as implemented by the CRONUS online calculator. Taking these effects into account strongly reduces the differences in erosion rates between the coarse and fine fractions, although it does not completely eliminates it (see [5] for an analysis of the meaning of the remaining discrepancies). To fully capture the effect of the grain-size dependency, all calculations in the present work were conducted using the range of erosion values obtained after applying the corrections reviewed above. The coarse fraction is delivered to the stream by downslope transport during the unearthing, and subsequent downslope transport and decay the corestones. The transport of this coarse fraction to the creeks is likely slower than the

transport of surrounding particles, and therefore, the concentration of ^{10}Be in the coarse fraction still remains a lower boundary estimate for surrounding soil erosion rates in the coves.

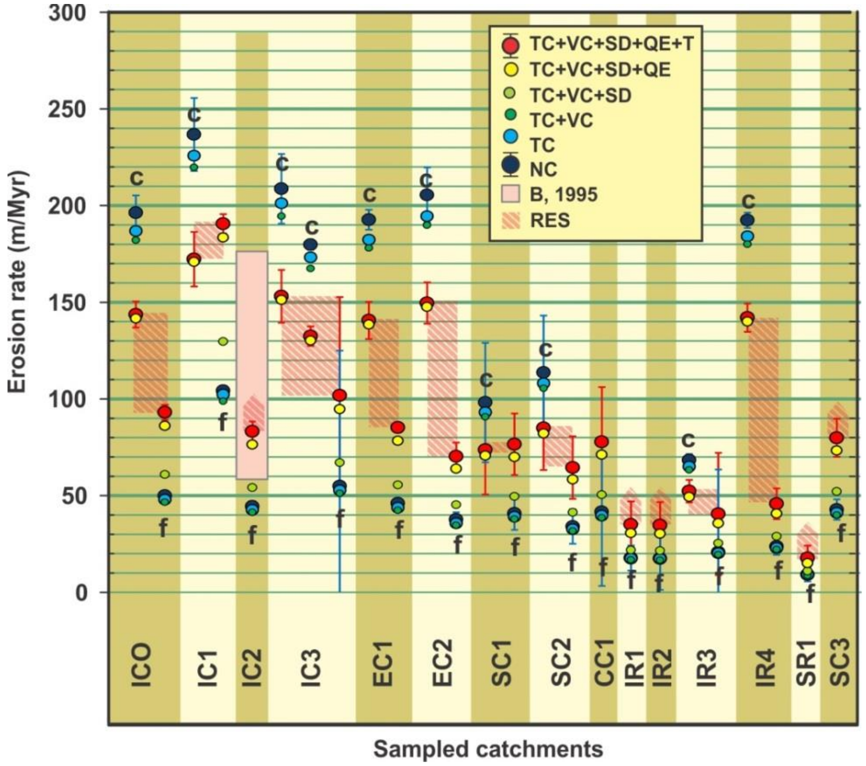

S3 - Figure 2. Sensitivity test of catchment-averaged denudation rates estimated from ^{10}Be concentration in the coarse and fine river sediment fractions after applying a suite of environmental corrections. NC: no correction, TC: topographic shielding correction, VC: vegetation shielding, SD: soil density, QE: quartz enrichment, T: time-dependence of production rates. B, 1995: spread of data in [4]. RES: residual spread in erosion rates between coarse and fine fractions after applying environmental corrections.

Stream	Grain size	NC ε_o	TC ε_o	TC+VC ε_o	TC+VC+DV ε_o	TC+VC+DV +QE ε_o	TC+VC+DV +QE+T ε_o
		(m/My)	*(m/My)*	*(m/My)*	*(m/My)*	*(m/My)*	*(m/My)*
CC1	*F*	*41 ± 3*	*40 ± 3*	*38 ± 2*	*50 ± 4*	*63 ± 4*	*69 ± 5*
EC1	*C*	*193 ± 17*	*183 ± 16*	*178 ± 16*	*138 ± 12*	*138 ± 12*	*141 ± 12*
	F	*46 ± 3*	*44 ± 3*	*42 ± 3*	*45 ± 3*	*70 ± 5*	*76 ± 5*
EC2	*C*	*206 ± 17*	*195 ± 17*	*190 ± 15*	*148 ± 12*	*148 ± 12*	*150 ± 12*
	F	*37 ± 3*	*36 ± 3*	*34 ± 2*	*45 ± 3*	*57 ± 4*	*63 ± 4*
IC0	*C*	*197 ± 23*	*187 ± 22*	*182 ± 21*	*142 ± 16*	*142 ± 16*	*144 ± 16*
	F	*50 ± 5*	*48 ± 5*	*46 ± 5*	*61 ± 7*	*77 ± 8*	*83 ± 9*
IC1	*C*	*237 ± 21*	*226 ± 20*	*220 ± 19*	*171 ± 15*	*171 ± 15*	*172 ± 15*
	F	*104 ± 12*	*102 ± 12*	*99 ± 11*	*130 ± 15*	*164 ± 19*	*171 ± 19*
IC2	*F*	*44 ± 3*	*42 ± 3*	*41 ± 3*	*54 ± 4*	*71 ± 5*	*78 ± 6*
IC3	*C*	*209 ± 17*	*202 ± 16*	*195 ± 15*	*151 ± 12*	*151 ± 12*	*153 ± 12*
	C	*180 ± 14*	*173 ± 14*	*168 ± 13*	*130 ± 10*	*130 ± 10*	*132 ± 10*
	F	*55 ± 4*	*54 ± 4*	*51 ± 4*	*67 ± 5*	*85 ± 6*	*91 ± 7*
IR1	*F*	*17 ± 1*	*17 ± 1*	*16 ± 1*	*21 ± 2*	*27 ± 2*	*31 ± 2*
IR2	*F*	*17 ± 1*	*17 ± 1*	*16 ± 1*	*21 ± 2*	*27 ± 2*	*31 ± 2*
IR3	*C*	*66 ± 5*	*65 ± 5*	*63 ± 5*	*49 ± 4*	*49 ± 4*	*52 ± 4*
	F	*20 ± 1*	*20 ± 1*	*19 ± 1*	*25 ± 2*	*31 ± 2*	*36 ± 3*
IR4	*C*	*187 ± 13*	*184 ± 13*	*180 ± 13*	*140 ± 10*	*140 ± 10*	*142 ± 10*
	F	*22 ± 2*	*23 ± 2*	*22 ± 2*	*28 ± 2*	*36 ± 3*	*41 ± 3*
SC1	*C*	*98 ± 7*	*93 ± 7*	*91 ± 7*	*70 ± 5*	*70 ± 5*	*73 ± 5*
	F	*40 ± 3*	*39 ± 3*	*34 ± 2*	*45 ± 3*	*62 ± 4*	*68 ± 5*
SC2	*C*	*114 ± 9*	*108 ± 9*	*105 ± 9*	*82 ± 7*	*82 ± 7*	*85 ± 7*
	F	*36 ± 2*	*33 ± 2*	*31 ± 2*	*41 ± 3*	*52 ± 4*	*57 ± 4*
SC3	*F*	*40 ± 3*	*41 ± 3*	*39 ± 3*	*51 ± 4*	*65 ± 5*	*71 ± 5*
SR1	*F*	*8 ± 1*	*8 ± 1*	*7 ± 1*	*10 ± 1*	*12 ± 1*	*15 ± 1*

S3 - Table 2. ^{10}Be-derived denudation rates for various correction parameters, using the coarse (c) and fine (f) sediment fractions as defined in Table 1. ε_o (NC): erosion rate in case of no shielding, for a vertically homogeneous soil density of 2.1 g cm^{-3}. TC: with topographic shielding, VC: vegetation shielding, DV: spatially-variable soil density (6) in S3 Table 4, QE: spatially-variable soil quartz enrichment (5) in S3 Table 4, and T: effect of time-dependent production rate estimated using the CRONUS calculator.

Rock/soil sample	Easting	Northing	Elevation	Quartz mass	[^{10}Be]	Shielding factor(1)	P_{μ} (2)	P_{spal}(3)	Quartz enrichment factor(4)	Integrated wet bulk density(5)	Erosion rate
	(°)	*(°)*	*(m)*	*(g)*	*(10^4 at g^{-1})*		*(at g^{-1} yr^{-1})*	*(at.g^{-1} y^{-1})*		*(g cm^{-3})*	*m My^{-1}*
CORS-ES0	-65.7962	18.2918	757	53.5	21.3 ± 0.5	0.927	0.23	4.73	1.4 ± 0.3	1.6± 0.2	51.1± 14
CORS-IC4	-65.7920	18.2763	750	51.5	14.9 ± 0.4	0.945	0.23	4.77	1.4 ± 0.3	1.6± 0.2	74.5 ± 20.9
CORS-IC5	-65.7928	18.2806	745	53.0	13.1 ± 0.4	0.945	0.23	4.75	1.4 ± 0.3	1.6± 0.2	84.7 ± 23.9
MNOK	-65.7775	18.2852	843	45.1	23.9 ± 0.5	0.925	0.24	5.03	1.4 ± 0.3	1.6± 0.2	47.6 ± 13.4
IC-PAR	-65.7854	18.2688	652	55.3	26.2 ± 0.5	0.928	0.22	4.38	1.4 ± 0.3	1.6± 0.2	38.7 ± 10.9
ET-SS	-65.8199	18.2780	880	48.0	19.8 ± 0.4	0.917	0.24	5.12	1.4 ± 0.3	1.6± 0.2	59.8 ± 16.8
ES-R1	-65.7963	18.2894	795	56.4	18.0 ± 0.5	0.940	0.23	4.93	1.4 ± 0.3	1.6± 0.2	62.8 ± 17.8
ES-R2	-65.8031	18.2899	795	46.1	18.9 ± 0.4	0.937	0.23	4.92	1.4 ± 0.3	1.6± 0.2	59.6 ± 16.7
ES-R3	-65.8053	18.2882	838	52.0	15.1 ± 0.4	0.940	0.24	5.09	1.4 ± 0.3	1.6± 0.2	77.1 ± 21.7
ES-R4S	-65.7985	18.2900	787	51.5	17.3 ± 0.3	0.940	0.23	4.90	1.4 ± 0.3	1.6± 0.2	65.2 ± 18.1
ES-R5	-65.7991	18.2889	792	50.7	17.9 ± 0.4	0.940	0.23	4.92	1.4 ± 0.3	1.6± 0.2	63.1 ± 17.7
SAB-R1	-65.7978	18.2780	664	51.9	28. 8± 0.5	0.928	0.22	4.42	1.4 ± 0.3	1.6± 0.2	35.3 ± 9.9
ES-COT1	-65.7992	18.2950	760	62.6	24.5 ± 0.4	0.929	0.23	4.75	1.4 ± 0.3	1.6± 0.2	44.3 ± 12.4
ES-COT2	-65.7978	18.2926	755	50.5	18.8 ± 0.5	0.929	0.23	4.73	1.4 ± 0.3	1.6± 0.2	58.2 ± 16.4
ES-COT3	-65.7971	18.2928	748	37.2	26.2 ± 0.6	0.928	0.23	4.70	1.4 ± 0.3	1.6± 0.2	40.9 ± 11.6
IC-PAT	-65.7881	18.2864	627	43.2	25.4 ± 0.5	0.927	0.22	4.30	1.4 ± 0.3	1.6± 0.2	39.3 ± 11.1
IC-COT1	-65.7906	18.2880	650	50.5	20.8 ± 0.4	0.926	0.22	4.37	1.4 ± 0.3	1.6± 0.2	49.2 ± 13.8
IC-COT2	-65.7850	18.2829	668	46.3	17.5 ± 0.4	0.929	0.22	4.44	1.4 ± 0.3	1.6± 0.2	59.7 ± 16.7
IC-COT3	-65.7900	18.2900	683	51.7	24.9 ± 0.5	0.929	0.23	4.49	1.4 ± 0.3	1.6± 0.2	41.6 ± 11.7
IC-COT3-SS	-65.7900	18.2898	681	50.6	22.1 ± 0.8	0.929	0.23	4.48	1.4 ± 0.3	1.6± 0.2	48.0 ± 13.8
IC-CLE	-65.7847	18.2792	655	44.6	26.3 ± 0.5	0.940	0.22	4.45	1.4 ± 0.3	1.6± 0.2	38.9 ± 11.0
FP-SAB-10	-65.7997	18.2794	661	50.8	66.0 ± 1.1	0.929	0.22	4.41	1.4 ± 0.3	1.6± 0.2	14.1 ± 4.1
PAC-SS	-65.7945	18.2931	750	58.4	17.2 ± 0.5	0.906	0.23	4.60	1.4 ± 0.3	1.6± 0.2	63.7 ± 18.0
PAC-10	-65.7945	18.2931	750	49.4	15.6 ± 0.5	0.906	0.23	4.60	1.4 ± 0.3	1.6± 0.2	68.0 ± 19.3

S3 - Table 3. ^{10}Be soil samples locations on broad shallow-sloping hilltops, ^{10}Beconcentrations and environmental parameters used for the calculation of denudation rates. [1]: Combination of topographic and vegetation shielding. [2,3] Production rates for neutrons (P_{spal}) and muons (P_{μ}), calculated using the CRONUS calculator for a polar sea-level ^{10}Be production rate of 5.1 at.g^{-1}.y^{-1}, for indicated shielding factor. [4] Quartz enrichment from averaging published measurements [11, 14]. [5] Average wet soil densities measured by White, Blum [11] and by the authors. *: soils from a narrow ridgeline, affected by unsteady erosion, excluded from the calculation of the average hilltop erosion rate of 49.7 ± 3.3 m My^{-1} inferred from the last column.

Stream	Grain size range(1)	Easting	Northing	Quartz(2) feeding area (A_Q)	A_Q Average elevation	Quartz mass	[^{10}Be]	Topographic shielding factor(3)	P_μ (4)	P_{spal}(4)	Quartz enrichment factor(5)	Integrated soil density(6)
	(ϕ) unit	*(°)*	*(°)*	*(km²)*	*(m)*	*(g)*	*(10^4 at g^{-1})*		*(at g^{-1} y^{-1})*	*(at.g^{-1} y^{-1})*		*(g cm^{-3})*
CC1	[4 3] f	-65.8055	18.2700	0.12	678	28.6	15.0 ± 0.4	0.93	0.21	4.24	1.4 ± 0.3	1.6± 0.2
EC1	[-3 -4] c	-65.7985	18.2922	0.01	773	59.6	3.63 ± 0.15	*0.92**	0.22	4.47	1.0 ± 0.0	2.7 ± 0.1
	[3 2] f					48.0	14.3 ± 0.4	0.93	0.22	4.52	1.4 ± 0.3	1.6± 0.2
EC2	[-3 -4] c	-65.7963	18.2909	0.03	773	36.6	3.4 ± 0.16	*0.92**	0.22	4.47	1.0 ± 0.0	2.7 ± 0.1
	[3 2] f					66.6	17.3 ± 0.4	0.93	0.22	4.52	1.4 ± 0.3	1.6± 0.2
IC0	[-4 -5] c	-65.7887	18.2869	0.13	706	32.1	7.36 ± 0.24	*0.92**	0.22	4.25	1.0 ± 0.0	2.7 ± 0.1
	[4 3] f					10.3	12.8 ± 1.0	0.94	0.22	4.35	1.4 ± 0.3	1.6± 0.2
IC1	[-1 -2] c	-65.7883	18.2904	0.29	725	37.0	2.90 ± 0.16	*0.92**	0.21	4.30	1.0 ± 0.0	2.7 ± 0.1
	[4 3] f					13.8	6.41 ± 0.53	0.97	0.22	4.53	1.4 ± 0.3	1.6± 0.2
IC2	[4 3] f	-65.7901	18.2821	0.11	707	27.6	14.3 ± 0.5	0.94	0.22	4.37	1.4 ± 0.3	1.6± 0.2
IC3	[-5 -6] c	-65.7888	18.2785	0.17	687	52.1	3.22 ± 0.15	*0.92**	0.21	4.22	1.0 ± 0.0	2.7 ± 0.1
	[-3 -4] c					42.7	3.72 ± 0.17	*0.92**	0.21	4.22	1.0 ± 0.0	2.7 ± 0.1
	[3 2] f					24.1	11.59 ± 0.45	*0.92**	0.21	4.22	1.0 ± 0.0	1.6± 0.2
IR1	[3 2] f	-65.7875	18.2801	0.02	665	49.0	32.9 ± 0.7	0.98	0.22	4.36	1.4 ± 0.3	1.6± 0.2
IR2	[4 3] f	-65.7905	18.2760	0.01	737	55.0	34.8 ± 0.8	0.99	0.22	4.63	1.4 ± 0.3	1.6± 0.2
IR3	[-3 -4] c	-65.7879	18.2717	0.06	691	42.7	9.43 ± 0.32	*0.92**	0.21	4.11	1.0 ± 0.0	2.7 ± 0.1
	[4 3] f					37.2	28.9 ± 0.7	0.97	0.21	4.21	1.4 ± 0.3	1.6± 0.2
IR4	[-3 -4] c	-65.7866	18.2703	0.22	641	49.3	3.40 ± 0.12	*0.92**	0.21	4.20	1.0 ± 0.0	2.7 ± 0.1
	[4 3] f					10.6	24.9 ± 0.9	0.95	0.22	4.41	1.4 ± 0.3	1.6± 0.2
SC1	[-4 -5] c	-65.8011	18.2844	0.24	724	50.4	6.79 ± 0.25	*0.92**	0.21	4.32	1.0 ± 0.0	2.7 ± 0.1
	[4 2] f					22.3	15.6 ± 0.5	0.94	0.22	4.41	1.4 ± 0.3	1.6± 0.2
SC2	[-2 -3] c	-65.7979	18.2802	0.15	699	18.1	5.83 ± 0.27	*0.92**	0.21	4.25	1.0 ± 0.0	2.7 ± 0.1
	[3 2] f					56.5	18.3 ± 0.4	0.94	0.22	4.34	1.4 ± 0.3	1.6± 0.2
SC3	[4 4] f	-65.7970	18.2778	0.004	652	16.8	14.6 ± 0.6	0.94	0.21	4.20	1.4 ± 0.3	1.6± 0.2
SR1	[4 3] f	-65.8000	18.2804	0.03	666	22.4	65.1 ± 1.1	0.99	0.22	4.41	1.4 ± 0.3	1.6± 0.2

S3- Table 4. ^{10}Be concentrations and environmental parameters used for the calculation of catchment-wide erosion rates

[1] Grain size phi scale of analyzed sediment (grain diameter $D = D_o\ 2^{-\phi}$ with D_o= 1 mm), used to define a coarse (c) and fine (f) fraction. [2] Extent of the quartz-diorite in each catchment. [3] Topographic shielding factor for the fine fraction is the average shielding in the quartz-feeding area. For the coarse fraction, it is the average shielding measured in corestone-strewn coves [4] Production rates for neutrons (P_{spal}) and muons (P_μ), calculated using the CRONUS calculator for a polar sea-level ^{10}Be production rate of 5.1 at.g^{-1}.y^{-1}, for the indicated topographic shielding and

vegetation shielding of 7%. [5] coarse fraction is assumed to be derived from corestones, and does not experience quartz enrichment. [6] ground density of the coarse fraction is the density of dense quartz diorite, while the density of the fine fraction is the average wet density of soil and of fully weathered saprolite.

Correction model	Average cove erosion rate		Hilltop erosion rate	Cove erosion rate as multiple of hilltop rate		Depth of incision of coves below ridges		Time of initiation	
	Coarse fraction	Fine Fraction		Coarse fraction	Fine Fraction	Coarse fraction	Fine Fraction	Coarse fraction	Fine Fraction
	m My^{-1}	*m My^{-1}*	*m My^{-1}*			*m*	*m*	*My*	*My*
NC	170	38	31	x 5.4	x 1.2	15	10	0.11	1.4
TC+VC+DV +QE+T ε_o	124	73	50	x 2.5	x 1.5	15	10	0.20	0.44

S3- Table 5. Average means values used for investigating the age of initiation of topographic differentiation, using the model with all environmental corrections (NC) and all performed corrections (TC+VC+DV +QE+T εo)

References

1. Kohl CP, Nishiizumi K. CHEMICAL ISOLATION OF QUARTZ FOR MEASUREMENT OF INSITU-PRODUCED COSMOGENIC NUCLIDES. Geochimica Et Cosmochimica Acta. 1992;56(9):3583-7.
2. Pett-Ridge JC. Contributions of dust to phosphorus cycling in tropical forests of the Luquillo Mountains, Puerto Rico. Biogeochemistry. 2009;94(1):63-80.
3. Reid EA. Characterization of African dust transported to Puerto Rico by individual particle and size segregated bulk analysis. Journal of Geophysical Research. 2003;108(D19).
4. Brown E, Stallard RF, Larsen MC, Raisbeck GM, Yiou F. Denudation rates determined from the accumulation of in-situ produced Be-10 in the Luquillo experimental forest, Puerto Rico.
. Earth and Planetary Science Letters. 1995;129(1-4):193-202.
5. Brocard GY, Willenbring JK, Scatena FN, Johnson AH. Effects of a tectonically-triggered wave of incision on riverine exports and soil mineralogy in the Luquillo Mountains of Puerto Rico. Applied Geochemistry. 2015;63:586-98.
6. Hidy AJ, Gosse JC, Pederson JL, Mattern JP, Finkel RC. A geologically constrained Monte Carlo approach to modeling exposure ages from profiles of cosmogenic nuclides: An example from Lees Ferry, Arizona. Geochemistry, Geophysics, Geosystems. 2010;11(9).

7. Brown ET, Stallard RF, Larsen MC, Bourlès DL, Raisbeck GM, Yiou F. Determination of predevelopment denudation rates of an agricultural watershed (Cayaguas River, Puerto Rico) using in-situ-produced 10Be in river-borne quartz. Earth and Planetary Science Letters. 1998;160(3-4):723-8.
8. Smith AL, Schellekens JH, Díaz A-LM. Batholiths as markers of tectonic change in the northeastern Caribbean. SPECIAL PAPERS-GEOLOGICAL SOCIETY OF AMERICA. 1998:99-122.
9. Fletcher R, Buss H, Brantley S. A spheroidal weathering model coupling porewater chemistry to soil thicknesses during steady-state denudation. Earth and Planetary Science Letters. 2006;244(1-2):444-57.
10. Chabaux F, Blaes E, Stille P, di Chiara Roupert R, Pelt E, Dosseto A, et al. Regolith formation rate from U-series nuclides: Implications from the study of a spheroidal weathering profile in the Rio Icacos watershed (Puerto Rico). Geochimica et Cosmochimica Acta. 2013;100:73-95.
11. White AF, Blum AE, Schulz MS, Vivit DV, Stonestrom DA, Larsen M, et al. Chemical weathering in a tropical watershed, Luquillo Mountains, Puerto Rico: I. Long-term versus short-term weathering fluxes. Geochimica et Cosmochimica Acta. 1998;62(2):209-26.
12. Orlando J, Comas X, Hynek SA, Buss HL, Brantley SL. Architecture of the deep critical zone in the Río Icacos watershed (Luquillo Critical Zone Observatory, Puerto Rico) inferred from drilling and ground penetrating radar (GPR). Earth Surface Processes and Landforms. 2016.
13. Porder S, Johnson AH, Xing HX, Brocard G, Goldsmith S, Pett-Ridge J. Linking geomorphology, weathering and cation availability in the Luquillo Mountains of Puerto Rico. Geoderma. 2015;249:100-10.
14. Ferrier KL, Kirchner JW, Riebe CS, Finkel RC. Mineral-specific chemical weathering rates over millennial timescales: Measurements at Rio Icacos, Puerto Rico. Chemical Geology. 2010;277(1-2):101-14.
